# Effect of Fluorescent Labels on DNA Affinity for Gold Nanoparticles

**DOI:** 10.3390/nano11051178

**Published:** 2021-04-29

**Authors:** Anna V. Epanchintseva, Ekaterina A. Gorbunova, Elena I. Ryabchikova, Inna A. Pyshnaya, Dmitrii V. Pyshnyi

**Affiliations:** Institute of Chemical Biology and Fundamental Medicine, SB RAS, Lavrentiev ave. 8, 630090 Novosibirsk, Russia; annaepanch@niboch.nsc.ru (A.V.E.); gorbunova-ekaterina@inbox.ru (E.A.G.); pyshnaya@niboch.nsc.ru (I.A.P.)

**Keywords:** oligonucleotides, fluorescent dye, gold nanoparticles, non-covalent adsorption, mechanisms, affinity, Langmuir constant, fluorophore importance

## Abstract

Fluorophore (FD) labeling is widely used for detection and quantification of various compounds bound to nanocarriers. The systems, composed of gold nanoparticles (GNPs) and oligonucleotides (ONs) labeled with FDs, have wide applications. Our work was aimed at a systemic study of how FD structure (in composition of ON-FDs) influenced the efficiency of their non-covalent associates’ formation with GNPs (ON-FD/GNPs). We examined ONs of different length and nucleotide composition, and corresponding ON-FDs (FDs from a series of xanthene, polymethine dyes; dyes based on polycyclic aromatic hydrocarbons). Methods: fluorometry, dynamic light scattering, high performance liquid chromatography, gel electrophoresis, molecular modeling and methods of thermodynamic and statistical analysis. We observed significant, differing several times, changes in surface density and Langmuir constant values of ON-FDs vs. ONs, evidence for the critical significance of FD nature for binding of ON-FDs with GNPs. Surface density of ON-FD/GNPs; hydrophobicity and total charge of ON or ON-FD; and charge and surface area of FDs were revealed as key factors determining affinity (Langmuir constant) of ON or ON-FDs for GNPs. These factors compose a specific set, which makes possible the highly reliable prediction of efficiency of ONs and ON-FDs binding with GNPs. The principal possibility of creating an algorithm for predictive calculation of efficiency of ONs and GNPs interaction was demonstrated. We proposed a hypothetical model that described the mechanism of contact interaction between negatively charged nano-objects, such as citrate-stabilized GNPs, and ONs or ON-FDs.

## 1. Introduction

A wide range of compounds, bound with different nanocarriers, are increasingly used in bioscience and biomedicine [1]. Quantification of these compounds binding to the carrier is necessary in many cases, and it can be carried out by methods without the use of labels, or using labels, which are mainly fluorophore dyes (FDs) [2,3,4,5,6,7,8]. “Typical” FDs contain conjugated aromatic rings and charged groups, and so are capable of interaction with different nanocarriers [9,10]. Nanocarriers vary considerably in size, chemical composition and hardness, and, in this huge set, gold nanoparticles (GNPs) are distinguished due to their spectral characteristics, non-toxicity, storage stability and relatively simple synthesis [11].

The amphiphilic nature of GNPs provides the ability of their surface to reversibly interact with surrounding molecules though electrostatic, hydrophobic and van der Waals forces [12]. Interaction of polar (charged) molecules with GNPs results in their polarization and in the formation of a heterogeneous electrostatic field around the GNP [13]. We think that long-range interactions should serve as factors that orient polar molecules relative to the GNP surface until the moment of their close contact. The adsorbed molecule is involved in a net of contact formation with the GNP surface through short-range interactions after binding, and this can lead to rearrangements in the secondary structure of the adsorbed molecule. It is obvious that the composition of an adsorbed molecule, especially one containing a fluorophore, should significantly affect the stability of the resulting associate.

The physico-chemical properties of GNPs and their covalent conjugates as well as non-covalent associates with various compounds, differing in their nature from simple citrate anions to peptides, nucleic acids and synthetic polymers, are quite well studied [14]. Both covalent and non-covalent binding provides the efficient and strong coating of GNPs with oligonucleotides (ONs) [15,16]; however, the latter have several attractive features [3,17]. Thus, ONs can be released from ON-GNPs (covalent conjugate of ON and GNP) only after breaking the covalent bond, and this can complicate or slow down the implementation of bioimaging or target action. At the same time, non-covalent adsorption facilitates the release of ONs from ON/GNP (non-covalent associate of ON and GNP), which should contribute to a more efficient implementation of bioimaging or targeted action. It also should be noted that obtaining ON/GNPs is easier than ON-GNPs [17]. For these and a number of other reasons, we have been studying non-covalent associates of GNPs with different ONs for a number of years [18,19,20].

Earlier we showed that the composition of ON is an important factor influencing the affinity of DNA sequences during competitive adsorption on GNPs. It turned out that heterogeneous G, T and A-rich sequences are mainly selected, while C-rich ONs disappear from the original randomized DNA pool. These results are in line with the well-known range representing the rising affinity of nucleotides to the gold carrier C < T < A < G [21].

At the same time, we proposed models of full or partial adsorption on GNPs of the native oligonucleotide, depending on its length [17]. Short oligonucleotides (6 mer) bind with GNP, forming the maximum number of short surface nucleotide/GNP contacts. Long oligonucleotides (26–40 mer) also bind to GNPs through short contacts, and the “excess” of nucleotides forms overhangs or loops on the GNP surface. The principles of partial adsorption of oligonucleotides on the GNP surface are described in [3,6].

Although fluorescence labeling of ONs is widely used, very little is known about the properties of labeled ONs bound with GNPs. Usually only one FD is introduced into the ON strand, and the affinity of this FD for GNP should be much stronger than those of all the nucleotides in the ON-FD composition. Many studies reported that covalent and non-covalent binding of ON-FDs to the GNPs leads to the crucial quenching of a dye fluorescence [22,23,24,25]. Fluorescence energy transfer causes this quenching from excited FD to GNP, which is most effective when FD tightly contacts with the GNP surface. Studies of the physico-chemical properties of ON-FD/GNPs and the effect of the nature of FD on these properties, as well as on the stability of associates, have not been published.

The aim of this work was to study the effect of some widely used fluorescent labels on the non-covalent adsorption of ON-FDs on GNPs. We characterized the binding of model oligothymidilate containing one of the xanthene dyes (Fluorescein (Flu) and Rhodamine B (RhB)); polymetine cyanine dyes (Cy3, Cy5, Cy7, Cy3.5, Cy5.5 and Cy7.5); and dyes based on polycyclic aromatic hydrocarbons (Pyrene (Pyr) and Perylene (Per)). We also examined other ONs and ON-FDs, which differed in length and nucleotide composition. We collected reference, experimental and simple computational data, which systematically characterized every ON-FD itself and the features of corresponding ON-FD/GNP. Different experimental and computational methods were applied in the study: fluorometry, dynamic light scattering (DLS), high performance liquid chromatography (HPLC) and gel electrophoretic analysis as well as the simplest molecular modeling approach and methods of thermodynamic and statistical analysis. We observed significant, differing several times, changes in the surface density and the Langmuir constant (*K_L_*) values of ONs containing different FDs. These results are evidence for the critical significance of FD nature for the binding of labeled ONs with GNPs. Key factors determining the affinity of ON-FD to GNP are charge and surface area of FDs; number of ON-FDs on one GNP; hydrophobicity of ON-FDs; and total charge of adsorbed ON-FDs.

This set of factors, experimentally determined or calculated based on generally known principles, allowed us to propose an approach, which makes possible the high-reliability prediction of the efficiency of ONs’ and ON-FDs’ non-covalent binding with GNPs. For the first time, a hypothetical model has been proposed that describes the mechanism of contact between negatively charged nano-objects, such as citrate-stabilized GNPs, and ONs or ON-FDs.

A full list of abbreviations used in the paper is shown in the Abbreviations section.

## 2. Materials and Methods

### 2.1. Chemicals

Tetrachloroauric acid (HAuCl_4_·3H_2_O) was purchased from Aurat PAO (Moscow, Russia). Tris and glycine were from Amresco Chemicals (Zottegem, Belgium). γ[^32^P]-ATP and T4 polynucleotide kinase (EC 2.7.1.78) were from Biosan (Novosibirsk, Russia). Fluorescein 5(6) and rhodamine B (mixed isomers) isothiocyanates and sodium citrate dihydrate (Na_3_C_6_H_5_O_7_∙2H_2_O) were purchased from Sigma-Aldrich (Darmstadt, Germany). Fluorescein phosphoramidite (6-isomer), FAM CPG (6-isomer), alkyne CPG modifier, TFA-amino modifier CPG, Cyanine3 NHS ester, Cyanine5 NHS ester, Cyanine5.5 NHS ester, Cyanine7 NHS ester, Cyanine7.5 NHS ester, pyrenebutyric acid NHS ester and perylene azide were from Lumiprobe (Moscow, Russia), dodecyl amine phosphoramidite was from NooGen (Novosibirsk, Russia). All fluorescent dyes (FDs) are presented in Table S1.

Water was purified by a Simplicity 185 water system Millipore (Burlington, MA, USA) and had a resistivity of 18.2 MΩ·cm at 25 °C. The deionized water was used to prepare buffers and oligonucleotide solutions.

### 2.2. Preparation of Oligonucleotides and Their Derivatives

Initial oligodeoxynucleotides (ONs) and their 5′-end-Flu-labeled derivatives were synthesized on an ASM-800 Biosset (Novosibirsk, Russia) by the solid-phase phosphoramidite protocol using phosphoramidites from ChemGenes (Wilmington, MA, USA). All synthesized ONs were purified by reversed-phase high-performance liquid chromatography (RP HPLC) on an Agilent 1200 Series using a Zorbax 5 μm Eclipse-XDB-C18 80 Å column (150 × 4.6 mm^2^), produced by Agilent (Santa Clara, CA, USA), with a linear gradient of acetonitrile in 0.02 M TEA-Ac (0–63%) over 30 min and an elution rate 1.5 mL per min.

ONs fluorescently labeled with 3′-End (Flu, RhB, Pyr, Cy3, Cy5, Cy5.5, Cy7 and Cy7.5) were prepared using [26]. T26 labeled with 3′-end Per was prepared using CuAAC click chemistry protocol [27]. Labeling of ONs with 5′-end-[^32^P] (T26, T26-Flu, T26-Per, T26-Cy5) was performed as described earlier [17]. Both 3′-end fluorescently labeled ONs and 5′-end-[^32^P]-labeled ONs were purified by electrophoresis in 15% polyacrylamide gel containing 8M Urea.

Concentrations of all ONs and ON-FDs were calculated using molar coefficients of absorption of the dinucleotides at wavelength 260 nm [28] and molar coefficients of absorption at wavelength 260 nm (***ε***) of the FD from suppliers’ web sites [29,30,31].

### 2.3. Molecular Properties of FDs

Structures of FDs (without linkers) were analyzed with the use of HyperChem molecular modeling system (HyperCube, Inc., Las Vegas, NV, USA). PM3 algorithm was applied to calculate partial charge of atoms and to optimize molecular structure of a dye. Solvent accessible surface of a dye was calculated using internal QSAR block of the software HyperChem (HyperCube, Inc., Las Vegas, NV, USA).

### 2.4. Hydrophobicity of ONs and ON-FDs

Hydrophobicity of ONs and ON-FDs were studied by RP HPLC Milichrom A-02 (Econova, Novosibirsk, Russia) using RP column ProntoSil 120-5-C18 (Econova, Novosibirsk, Russia) with gradient elution of acetonitrile (0–61.2%) in 0.02 M TEA-Ac (pH 6.0) over 17 min and an elution rate 0.2 mL per min. The hydrophobicity coefficient (*H*) was determined according to the equation:

*H* = (*tr*_(ON)_ − *tr*_0_)/*tr*_0_, (1)

where *tr*_(ON)_ is retention time of ONs or ON-FDs (min) and *tr*_0_ is dead time of column under condition used (min) [32,33,34].

### 2.5. Preparation and Characterization of Citrate-Coated GNPs

GNPs were prepared using a classic citrate reduction procedure [35]. The size and monodispersity of suspension of GNPs were determined by (1) transmission electron microscopy (TEM). Suspension of GNPs was adsorbed on copper grids covered with formvar film for 30 s and examined in a JEM 1400 TEM (Jeol, Tokyo, Japan) supplied with a Veleta digital camera (EM SIS, Muenster, Germany). Size of GNPs was determined directly on the digital camera screen using the camera’s software. Examination of GNPs in TEM revealed a homogeneous suspension consisting of spherical NPs (Figure S1) of 12.7 ± 2.0 nm in diameter.

DLS study using a Zetasizer Nano NS (Malvern Instruments, Worcestershire, UK) was applied for basic characterization of citrate-stabilized GNPs. Measurements were taken according to the equipment manufacturer’s instructions. The GNPs had a hydrodynamic diameter of 17.3 ± 2.1 nm, zeta-potential (*ζ*) of −33.6 ± 2.0 mV and polydispersity index (PdI) of 0.127 ± 0.020.

The concentration of GNPs was (3.6 ± 0.5) × 10^−9^ M, as calculated from absorbance at 520 nm using extinction coefficient (8.78 ± 0.06) × 10^8^ M^−1^ cm^−1^ [36].

### 2.6. Preparation of All ON/GNPs and ON-FD/GNPs

Preparation of all ON/GNPs and ON-FD was performed as described earlier [17]. Suspensions (final volume was 1.4 mL) containing GNPs and a 1−200-fold excess of ON or ON-FD were incubated for 24 h at 56 °C unless otherwise specified, sedimented for 30 min at 14,000 rpm and supernatants were collected. The activity of [^32^P]-containing specimens was counted in water using a liquid scintillation counter RackBeta1209 (Pharmacia/WallacOy, Turku, Finland). The fluorescence intensity of ONs labeled with Per, Pyr, Flu, RhB, Cy3, Cy5 and Cy5.5 was measured using a Clariostar plate fluorimeter (BMG Labtech, Ortenberg, Germany) and the fluorescence intensity of ONs labeled with Cy7 or Cy7.5 was measured using a Clariostar Plus plate fluorimeter (BMG Labtech, Ortenberg, Germany).

Before loading onto 0.8% agarose gel, 1 μL of 50% glycerol was added into samples as a loading solution. Electrophoresis was carried out for 10–90 min at 5 V/cm in 25 mM Tris and 250 mM glycine buffer, pH 8.3. Hydrodynamic diameter and zeta-potential of the prepared associates were determined using a Zetasizer Nano NS (Malvern Instruments, Malvern, UK). All measurements were taken at least in triplicate.

### 2.7. The Langmuir Isotherm

The concentration of each adsorbed ON was determined at a fixed concentration of GNPs (0.5 nM); a 1−200-fold excess of ON was added. The Langmuir constant was determined by fitting the data to the Langmuir isotherm:

*n* = *a* × *K_L_* [ON]/(1 + [ON] *K_L_*), (2)

where *n* is the adsorbed ON (molecules per GNP), *K_L_* is the Langmuir constant in M^−1^, a is the adsorption capacity (molecules per GNP) and [ON] is the ON molar concentration [16].

### 2.8. An Equilibrium Dissociation Constant

[^32^P]-labeled ONs (0.03–0.05 nM final concentration) were added to GNPs (to 0.03–3 nM final concentration) to a 1.4 mL final volume, incubated for 30 min at 56 °C and centrifuged for 30 min at 14,000 rpm; then, supernatants were collected. The radioactivity of supernatants was measured, and the concentration of unbound ON was determined. K_D_ values were determined by nonlinear fitting of parameters using the equation:

*Y* = *B_max_*[GNP_0_]/(*K_D_* + [GNP_0_]), (3)

where *Y* is the bound fraction of ON, [GNP_0_] is the total concentration of GNPs and *B_max_* is the number of binding sites [37].

This model was designed to analyze only the specific binding of the ligand to the target. Under the conditions of the described experiment, nonspecific binding was excluded. Nonlinear regression analysis was carried out with the use of GraphPad Prism 5.04 (GraphPad Software, Inc., San Diego, CA, USA, 2010). “Typical” graph is shown on Figure S2.

### 2.9. ON Surface Density

ON surface density (number of ON molecules on one GNP) was determined on the base of fluorescence intensity and radioactivity, according to the equation:

*n* = [ON_b_]/[ON_0_], (4)

where *n* is the ON surface density, [ON_b_] is the concentration of ONs associated with GNPs and [ON_0_] is the total concentration of GNPs.

### 2.10. Stability of All ON/GNPs and ON-FD/GNPs

Stability of all ON/GNPs and ON-FD/GNPs in the same agarose gel was studied by band blurring (during electrophoresis) and band diffusion (without electric field).

#### 2.10.1. Blurring of Bands during Electrophoresis

**Figure 1:**
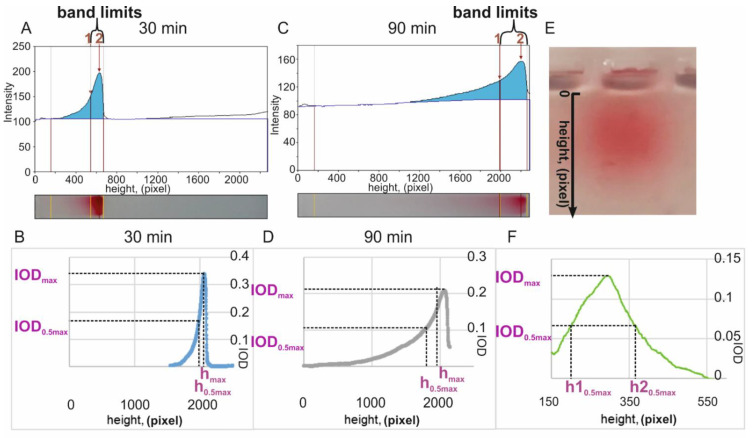
Analysis of agarose gels containing ON/GNPs. Optical density profiles for blurring analysis (**A**,**C**), by Gel Analyzer, and for Δ*h* analysis (**B**,**D**), by Gel Pro, after electrophoresis for 30 and 90 min. Gel scans corresponding to the gel profiles are shown at the bottom of figures A and C. Diffusion of ON/GNPs in agarose gel: gel scan (**E**) and optical density profile (**F**), by Gel Pro after 96 h diffusion.

Scanned images of agarose gel were obtained after 30 and 90 min of electrophoresis; the images of the bands in gel were analyzed using the programs Gel Analyzer 19.1 (www.gelanalyzer.com, created by Istvan Lazar Jr., PhD, and Istvan Lazar Sr., PhD, CSc) (Figure 1A,B) and Gel–Pro Analyzer v.4.0.00.001 (Media Cybernetics, L.P., Rockville, MD, USA, 2000) (Figure 1C,D).

Band blurring (*bl*) was evaluated using the following formula:

(5) $$bl=\frac{\;\mathrm{IOD}\left(30\;\min\right)}{\mathrm{IOD}\left(90\;\min\right)}\times 100\%,$$

where IOD(30 min) and IOD(90 min)–the total optical density of the band after 30 min and 90 min of electrophoresis, respectively (band limits on Figure 1A,B).

Δ*h*-analysis was evaluated using the following formula:

(6) $$\Delta h=\frac{h\max\left(90\;\min\right)-h0.5\max\left(90\;\min\right)}{h\max\left(30\;\min\right)-h0.5\max\left(30\;\min\right)},$$

where *h*max(90 min) and *h*max(30 min)–distance (in pixels) from the gel pocket to the position of the area with maximum optical density in the band (IOD_max_, Figure 1C,D) and *h*0.5max(90 min) and *h*0.5max(30 min)–distance (in pixels) from the gel pocket to the position of area with half the maximum optical density of the band (IOD_0.5max_, Figure 1C,D).

#### 2.10.2. Diffusion of Bands without Electric Field

Electrophoresis was stopped after 10 min (for the associates to enter the gel) and the gel was left at room temperature and scanned after 0, 3, 6, 9, 25 and 96 h. The images of the gel bands were analyzed using the program Gel Pro (Figure 1E,F). The degree of band passive diffusion (*pd*) was calculated for different time (*t*) points by the formula:

*pd*(*t*) = (*h*2_0.5max_(t) − *h*1_0.5max_(*t*))/(*h*2_0.5max_(0) − *h*1_0.5max_(0)), (7)

where *h*2_0.5max_–distance (in pixels) from the gel pocket to the lower position of half of the maximum optical density of the band in the gel (IOD_0.5max_, Figure 1C,D); *h*1_0.5max_–distance (in pixels) from the gel pocket to the upper position of half of the maximum optical density of the band in the gel (IOD_0.5max_, Figure 1D,F).

### 2.11. Nonlinear Regression

All obtained data were taken at least in triplicate, and obtained data sets were analyzed using GraphPad Prism 5.04 (GraphPad Software, Inc., San Diego, CA, USA, 2010). Data were fitted to the Langmuir isotherm or binding curve. Standard deviation is represented on graphs as error bars.

### 2.12. Statistics

Results obtained by methods described in Section 2.7, Section 2.8, Section 2.9 and Section 2.10 were statistically processed using Student’s test, and values of *p* ≤ 0.05 were considered to be statistically significant. Microsoft Excel software (Microsoft Corporation, Redmond, WA, USA, 2016) was used for analysis. Data are expressed as means Std.Err. for at least three independent experiments. Multiple regression analysis was carried out using Statistica Ultimate Academic 13.3 software (Statsoft, Tulsa, OK, USA, 2021) which was applied to reveal statistically significant energy increments determining efficacy of binding ONs.

## 3. Results

### 3.1. Background Information

**Figure 2:**
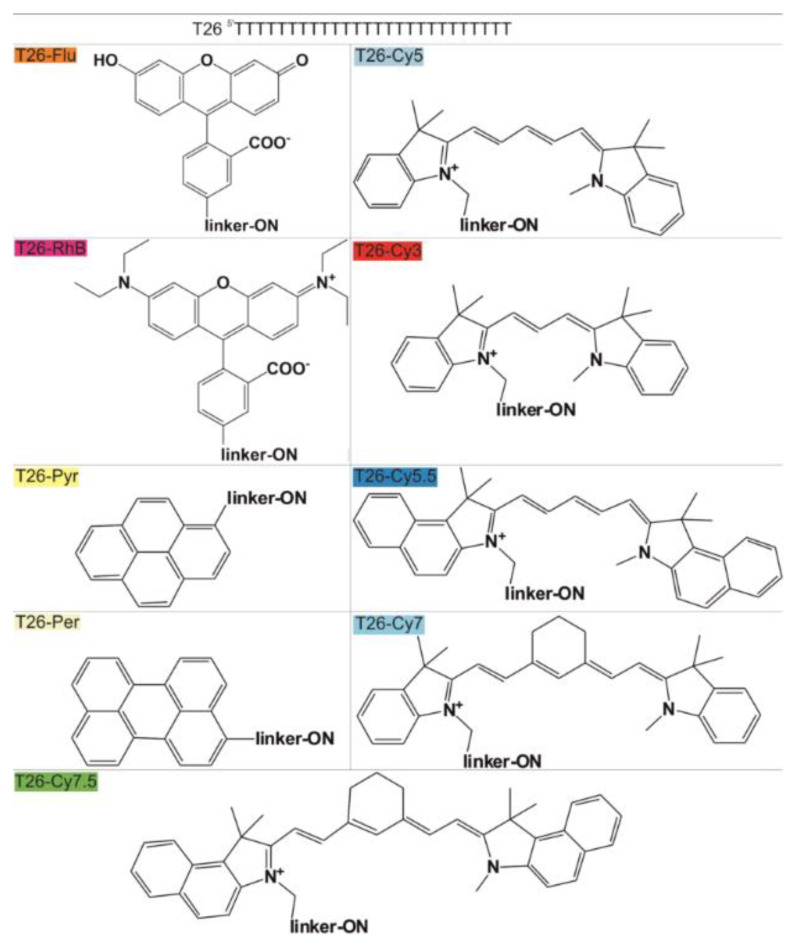
Designation of fluorescently labeled oligothymidylates. Complete formulas of FDs and linker fragments are presented in Table S1. The color of the boxes with T26-FD names corresponds to color of FD solution in water at pH 5.5.

Fluorescent labels of ONs are widely used to assess the efficiency of ON adsorption (covalent or non-covalent) on various surfaces, including GNPs [2,3,4,5,6,7,8]. A question arises: could a structure of FDs influence the adsorption of labeled ONs to the surface of GNPs? To know that, we prepared and examined a set of derivatives of 26-mer homothymidalate (T26) containing various fluorescent dyes (T26-FDs) (Figure 2). We chose widely used FDs: (i) xanthene dyes Fluorescein (Flu) and Rhodamine B (RhB); (ii) polymetine dyes Cy3, Cy5, Cy7, Cy3.5, Cy5.5 and Cy7.5; and (iii) dyes based on polycyclic aromatic hydrocarbons, Pyrene (Pyr) and Perylene (Per). The structure of fluorescent dyes is presented in Table S1. Our previous studies revealed that incubation conditions, length and composition of ONs influence the kinetics and affinity of their non-covalent adsorption to GNPs surface [17]. In this study, mixtures of GNPs and all the above-listed ONs were incubated under the same conditions, which provided effective non-covalent adsorption of ONs to the surface of GNPs (17.3 ± 2.1 nm in diameter, DLS data) [21].

The T26 does not form intra- and intermolecular structures, and so it was chosen as a model compound to exclude the possible influence of spatial organization features of the ON chain on the efficiency of ON-FD/GNP formation [38]. Using T26 with a fixed length and nucleotide composition, we were able to investigate the effect of different FDs on the properties of ON-FD/GNPs, including ON adsorption to surface of GNPs. Radioactively labeled T26 (pT26) served as a control.

To analyze the effect of FD structure on the efficiency of the corresponding T26–FD interaction with GNPs, we used our own experimental data as well as published physicochemical characteristics of the FDs (Table S1).

Using HyperChem software, the surface area and mass of the FDs were calculated. The linker fragment between T26 and FD is unable to absorb and emit fluorescence, so its structure (Table S1) was not taken into account in these calculations.

The total charge of the dye itself, and the charge of an additional unit of the phosphodiester linker providing FD attachment to ON, were taken as the value of the FD charge at pH 5.5 (the conditions of obtaining ON-FD/GNPs) (Table S1).

To establish patterns and features of the interaction of ON-FDs with the GNP surface, we used reference, calculated and experimental data.

### 3.2. Adsorption of Fluorescently Labeled ONs to GNPs

We prepared T26 labeled with all used FDs and examined adsorption of the resulting T26-FDs to GNPs at a fixed concentration of GNPs (0.5 nM) and increasing concentrations of T26-FDs (0.5–100 nM). Radioactively labeled T26 served as the original control.

**Figure 3:**
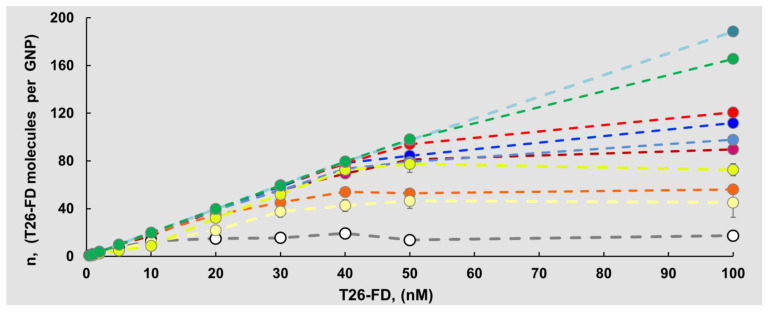
Langmuir isotherms determined in the process of adsorption of T26 и T26-FDs on GNPs (at 0.5 nM concentration of the GNPs). The color of the curve matches the color of the boxes with T26-FD names, “color” means color of FD solution in water at pH 5.5.

Unexpectedly, we detected that the presence of FDs in ON lead to a significant increase in the efficiency of ON adsorption to GNPs: *K_L_* = 0.147 μM^−1^ (native T26) and *K_L_* ≈ 0.019 µM^−1^ (T26-FDs, value was averaged over the set of FDs used). The saturation curves (Langmuir isotherms) of all ON-FD/GNPs associates are shown in Figure 3.

The presence of Flu, which is one of the “small” dyes in size (T26-Flu), resulted in a *K_L_* decrease by a factor of 3.4, which was the minimum significant decline. At the same time, addition of Cy7 (one of largest FDs) to T26 reduced the *K_L_* by 190 times, and this drop was accompanied with an increase in the number of T26-Cy7 molecules effectively binding to the surface of one GNP (Figure 3, Table 1).

In general, the presence of a terminal FD residue in ON increased the maximum achievable amount of ON molecules capable of effectively adsorbing on GNP by more than six times, and this amount depended both on *K_L_* values and fluorophore structure. One GNP adsorbs only approximately 17 molecules of fluorophore-free T26, while in the case of T26-Pyr, the same particle is capable of adsorbing approximately 45 molecules of T26-Pyr, that is, 2.7 times more. This contribution to the increase in the adsorption density of T26-FD on the GNP surface was the minimum, and the Pyr residue was one of the smallest FDs. The greatest increase in the number of adsorbed T26-FD molecules on GNP, by more than 11 times (up to ~189 molecules), was observed for T26 carrying a large residue Cy7 (Table 1). Analysis of the data showed that the introduction of any FD residue (comparable by size to one nucleotide) into the structure of T26 lead to almost 8-fold decrease in the value of the *K_L_* (Table 2).

To compare the effects caused by the introduction of FD and elongation of the nucleotide part, we used another set of ON derivatives. We extended 20-mer N*-Flu (Table 2) from the 5′-end by the fragments TTTTTT (N*1-Flu) or TTGTTG (N*2-Flu), and compared the values of *K_L_* and *n*, which characterize the efficiency of the formation of associates with GNPs. It was previously shown that both these fragments have an increased affinity for the GNP surface [21]. However, the introduction of these rather large nucleotide fragments (Δ*M_w_* ~1800 Da or 30% elongation) practically did not affect the adsorption parameters of the labeled ONs N*1-Flu and N*2-Flu on GNPs (Table 2).

To characterize the effect of FD chemical nature in the composition of the T26-FD on its affinity for GNPs in detail, we determined the equilibrium dissociation constant (*K_D_*) under conditions of the absence of ON’s intermolecular interaction during adsorption to the GNP surface. In this case, the T26 was simultaneously labeled with 5′-[^32^P] and 3′-FD residues. Binding of ON-FDs was performed at a constant concentration of ONs (~0.04 nM) and an increasing concentration of GNPs (0.03–3 nM). We used one FD from each series: (i) xanthene dyes: Flu; (ii) polymetine dyes: Cy5; and (iii) dyes based on polycyclic aromatic hydrocarbons: Pyr. The *K_D_* values decreased in the series: ON-Cy5 < ON-Pyr < ON-Flu. This dependence fully correlated (*R^2^* = 0.985) with the change in the *K_L_* values determined for corresponding T26-FDs. These data confirm that a change in dye structure significantly affects the stability of T26-FD/GNPs.

It is interesting that the effects associated with the presence of a fluorophore play a greater role than the change in ON length. The data obtained convincingly show that the presence of any FD (average *M_w_* (FD) ~400 Da) in ON-FD turned out to be unexpectedly significant. FD noticeably changed the affinity of a rather large ON (*M_w_* (T26) ~7800 Da) to GNPs, and the scale of changes obviously was determined by the structure of the introduced polyaromatic fluorescent residue. We believe that contact interactions of FD with the GNP surface are the critical factor determining the stability of non-covalent ON-FD/GNP associates.

Few previous publications noted that the presence of a fluorophore in ON might affect its affinity for GNP [39,40]; however, such effects have not been studied systematically. Meanwhile, it should be noted that significant changes in ON length (from 6-mer to 40-mer) can significantly alter the efficiency of ON/GNP formation, primarily due to changes in the topology of the nucleotide layer on the GNP surface [17]. The elongation of homothymidilates, for example, from 26 to 40 mers (i.e., by one and a half times) leads to an increase in the affinity of these ONs for GNP by a third, and an almost proportional decrease in their binding density on a nanoparticle by a factor of 3.3.

### 3.3. Spectrophotometric and Spectrofluorometric Analysis of T26-FD/GNPs Associates

GNPs have an exceptionally high extinction coefficient at maximum absorption in the visible region relative to T26 labeled with any FD (8.78 ± 0.06) ×10^8^ M^−1^ cm^−1^ [36]. Therefore, the spectra of associates can be hardly noticeable, even under the conditions of the maximum attainable loading for the T26-FDs and after washing of T26-FD/GNPs from unbound T26-FDs. These features determine the shape of the optical absorption spectra of all used T16-FD/GNPs, which are close to each other (Figure S4). Spectrofluorometric analysis of all T26-FD/GNPs could not provide detectable fluorescent signals from any associate (data are not shown). Such quenching of the dye fluorescence is possible only if the fluorophore is close to the GNP surface.

Based on published data on the quenching of ON-FDs bound to GNPs [41], we assume that when the FD residue shifts away from GNP, for example, at a distance comparable to the length of 26-mer ON (~7–8 Å), the efficiency of quenching may decrease and fluorescence may be detected.

### 3.4. Hydrodynamic Size and Net Charge of T-26-FD/GNPs

We measured, using the DLS method, hydrodynamic diameter (*d_H_*) and zeta potential (*ζ*) (representing effective net charge of T26-FD/GNPs) for each associate maximally loaded with ON-FDs (Table S2). Starting GNPs, stabilized with citrate only, had *d_H_* and ζ values of 17.3 ± 2.1 nm and −30 ± 4 mV, respectively. Citrate molecules on the GNP surface are able to be exchanged for ON-FD molecules, having a greater affinity for GNPs. Negatively charged ONs are adsorbed on GNPs forming stable non-covalent associates [25]. This process is well illustrated by the data obtained in this study (Table S2).

The native T26 forms T26/GNPs associate with a minimum surface density of the ONs (*n* = 17 ± 5). The hydrodynamic size of this associate somewhat increases (*d_H_* = 26 ± 0.3 nm), while the net charge practically does not change (ζ = −30.5 ± 1.6 mV) compared to starting GNPs. The surface density of T26 molecules labeled with polycyclic aromatic hydrocarbons and xanthene residues on GNPs on average was *n* ~ 59 and 70 molecules, respectively. Polymethine dyes with an increase in residue size show a tendency to form compact associates with a relatively high negative surface charge. The presence of Cy7 and Cy7.5 residues in the T26-FD structure resulted in the formation of relatively small particles (average *d_H_* ~ 28 nm) having a high net charge due to the extremely high number of ON-FDs on the GNP surface (average *n* ~ 177).

Thus, it can be stated that the presence of FD in the composition of T26 increases the surface density of the T26-FD/GNPs associate and increases its size (average *d_H_* ~ 31.2 nm) and the value of the effective net charge (average ζ ~ −39.2 mV).

### 3.5. Electrophoretic Analysis of Non-Covalent Adsorption of T26-FDs on GNPs

Electrophoretic separation of reaction mixtures in an agarose gel is a quick and convenient way to visualize the formation of ON-FD/GNPs. The method allows us to determine the regimes of concentration that provide GNP saturation and to characterize the homogeneity of ON-FD/GNPs [17,21,42]. The method also allows us to estimate the relative stability of the associates under nonequilibrium conditions of their movement through the gel matrix under the influence of an electric field.

Stable associates under electrophoresis conditions, while maintaining their structure, should also preserve clear contours of the band corresponding to the migration zone of full-size associates in the gel during the experiment. On the contrary, migration zones of unstable associates should be significantly more blurred.

**Figure 4:**
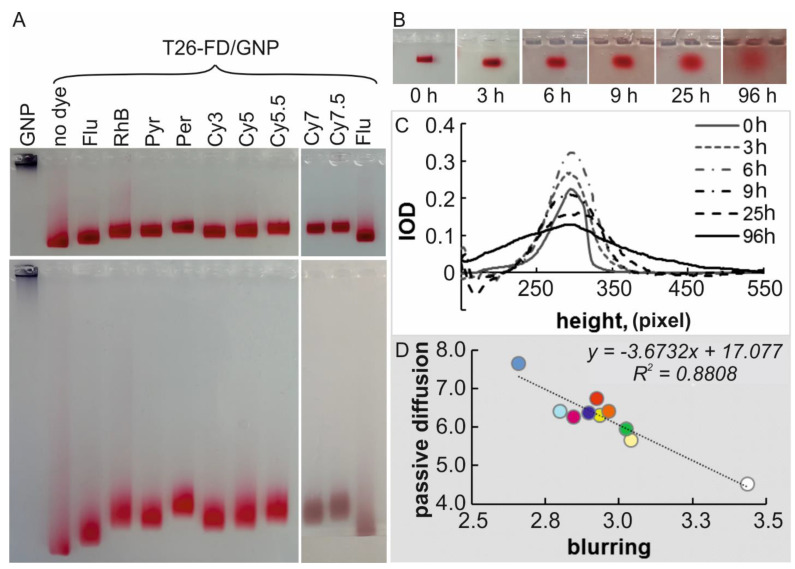
(**A**) Agarose gel electrophoresis of T26-FD/GNPs after 30 min (top) and 90 min (bottom). Associate names are indicated above the tracks. (**B**,**C**) Gel images of T26/GNPs, and curves obtained by Gel Pro software characterizing these images. (**D**) Correlation between passive diffusion of T26-FD/GNPs (without an electric field) and their blurring (in the presence of a field).

Comparative electrophoretic analysis of T26-GNPs and T26-FD/GNPs (using all FDs) showed that both the relative migration mobility of associates with maximum surface density in the gel, and the efficiency of baseband zone blurring, depend significantly on the presence of FD and its nature (Figure 4A).

The maximum decrease in electrophoretic mobility (*µ*) of T26-Per/GNPs was 17% (at marginal points) relative to the corresponding value for T26-GNPs (Table S2). The minimum relative changes in *µ* were observed for T26-Flu/GNPs, the only one of those studied, that contained negatively charged fluorescent residue under electrophoresis conditions, and had a surface density of ~55 T26-Flu molecules per one GNP. The largest slowdown in the migration rate of T26-FD/GNPs in the gel was detected for large positively charged polymethine residues of the Cy series (~13%). It is worth recalling that the introduction of Cy residues into T26 provided the highest surface density of T26-FDs on the GNP surface (Table 1).

The blurring of the main migration zones of T26-FD/GNPs may result not only from the diffusion of “filled” associates in the gel during electrophoresis, but may also reflect the process of partial desorption of T26-FDs from the GNP surface. Partially covered associates, resulting from desorption, always have lower *µ* values than “filled” ones, while “naked” GNPs (covered only with citrate), have a *µ* value tending to zero (Figure 4A, line 1—GNP).

The associates formed by native T26 and T26-FDs containing aromatic hydrocarbon residues (Pyr and Per), xanthene dyes (Flu and RhB) and the minimal Cy3 residue from the Cy series were most prone to blurring of migration zones. Polymethine residues Cy5, Cy5.5, Cy7 and Cy7.5 showed the greatest stability of T26-FD/GNPs during electrophoresis—the blurring of the migration zones of these associates was minimal. There is a fairly good correlation between the values of T26-FD/GNPs zeta potential, and the efficiency of associate zone blurring in the gel during electrophoresis.

To exclude the influence of the electric field, we studied the passive diffusion of T26-FD/GNPs in an agarose gel for 96 h at 25 °C (Figure 4B). These experiments demonstrated the diffusion rate of the NPs themselves in gel without the application of external forces, which made it possible to assess the mobility of T26-FD/GNPs, reflecting both the relative efficiency of NPs’ interaction with the gel matrix and their stability over time in a given medium.

It can be seen that, in all cases, the zones of GNPs in the gel were blurred in time, and all samples retained the color typical for each T26-FD/GNP associate. These results indicate a fairly high stability of associates and indicate that the efficiency of passive diffusion of T26-FD/GNPs in agarose differed quite significantly. We also found a significant anticorrelation (*R^2^* = 0.881. Figure 4D) between the efficiency of blurring zones of associates during migration under an electric field, and the rate of zones blurring during passive diffusion in agarose.

Thus, two pretty simple experiments clearly demonstrated differences in T26-FD/GNPs characteristics. In the case of passive diffusion (without electric field), the interaction of T26-FD/GNPs with the agarose matrix probably plays a greater role than under nonequilibrium conditions (in electric field) of electrophoretic migration of the associates in the gel. Analysis of the data shows that the denser the coverage of the GNP surface with ONs or ON-FDs, the less likely the interaction of electroneutral chains of the polysaccharide (agarose) with free areas of the GNP surface. This leads to an acceleration of passive migration of particles and a greater blurring of the associate localization zones in time. This is additionally confirmed by the fact of a pronounced interaction of “naked” GNPs stabilized with citrate with agarose.

### 3.6. Analysis of Relationship of ON-FDs Hydrophobicity and Their Affinity for GNP

We examined a set of T26-FD/GNPs, in which the fluorophores differed by (1) size of the conjugated π-system; (2) presence of exocyclic substituents and conjugated double bonds; and (3) charge of both FD itself and the linker between FD and ON. As a rule, an increase in the number of these structural features in a T26-FD molecule leads to an increase in its hydrophobicity.

We estimated the changes in hydrophobic properties of the studied T26-FDs using reversed-phase high-performance liquid chromatography (RP HPLC) on a column packed with a C18 phase (Figure S5A). The relative hydrophobicity of each T26-FD was determined in accordance with its retention time (tr) on the column (Figure S5B) [34].

As might be expected, the introduction of all FDs into the T26 lead to significant increase in the hydrophobicity of the T26-FDs. It should be noted that an increase in ON-FDs hydrophobicity correlated with an observable increase in its affinity for the surface of GNPs. The fixed length and composition of the nucleotide part (T26) allow us to say that more hydrophobic residues have a higher adsorption capacity to GNPs.

For example, most hydrophobic T26-FDs containing residues of Cy-series demonstrate a strong affinity to GNPs. Native T26, as well as T26-FDs containing residues of xanthene or aromatic hydrocarbon types, are less hydrophobic, and show a lower affinity for the GNP surface. We obtained reliable linear correlations (*R*^2^ ~0.72) between the tr and *K_L_* values for all T26-FD/GNPs associates, and the correlation of *tr* and *n* values also was determined for the full set, including T26/GNPs and T26-FD/GNPs (Figure S5B).

It should be noted that the hydrophobicity of T26-FDs is one of the most reliably determined experimental parameters. The range of changes in hydrophobicity for all analyzed associates (*tr* values) varied from 6.4 (for native T26) to 14 min (T26-Cy7.5). Thus, we have one more piece of evidence that the nature and physicochemical properties of FD residues covalently bound to an ON influence its affinity for GNPs.

All fluorescently labeled ONs used in this work carried structurally related planar unsaturated polyaromatic fluorophores, which made it possible to compare their hydrophobicity. Therefore, we analyzed the hydrophobicity and affinity of fluorescein-labeled ONs of the type Flu-(D)k-Tm for GNPs (Table 2). (D)—saturated dodecyl residue based on a phosphodiester of an N-substituted diethanolamine fragment, which replaces the corresponding amount of thymidine residues in T26 (Figure S5). The structure of these compounds was designed to keep the number of phosphate residue charges in ON, so, the number of D-residues (k) corresponded to 26 mer—Tm [43,44,45].

Our experiments showed that the introduction of one or two D residues (Flu-D-T25 and Flu-DD-T24) led to the expected decrease in *K_L_* and an increase in the surface density of the corresponding associates. However, additional hydrophobization of the ON, by the introduction of a third D residue (Flu-DDD-T23), led to an undesirable drop in its affinity for GNPs (Figure S6). We believe that this drop was due to the aggregate state of Flu-DDD-T23, because the conjugate (under concentration conditions for associates obtaining) is able to form self-aggregates with an average diameter close to 456 nm (DLS, data not shown). These conditions were required to achieve plateau values for GNP surface coverage. Other ONs of this series (Flu-D-T25 and Flu-DD-T24) did not form any aggregates, which is in good agreement with our previous observation [44].

Thus, the hydrophobicity of ON-FDs is one of the factors that determine the efficiency of their adsorption on the GNP surface. However, a more important factor seems to be the nature of the hydrophobic component. For example, a pair of Flu-DD-T24 and T26-Cy7 have similar retention times on an RP HPLC column: 12.6 and 11.8 min, respectively (Figure S6). At the same time, these derivatives differed greatly in their binding characteristics. The presence of an extensive aromatic system in the case of T26-Cy7 resulted in the largest GNP coverage (*n* ~189). Long aliphatic residues in Flu-DD-T24 were able to increase the affinity for GNP compared to parental Flu-T26 (*n* ~ 66 and 49, respectively), but could not reach the exceptional level demonstrated by its Cy7 partner (T26-Cy7) (Figure S6). The same observations were made after analyzing the relationship between the values of hydrophobicity and the *K_L_* for both series of samples T26-FDs and Flu-(D)k-Tm.

### 3.7. Correlation Analysis

**Figure 5:**
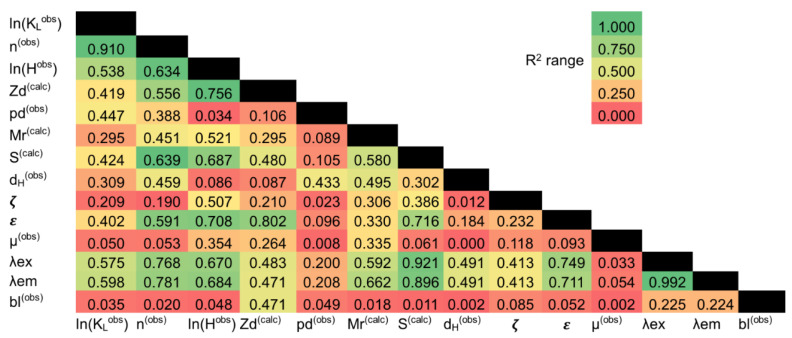
Hitmaps demonstrating the mutual correlation of Pearson’s criteria between the characteristics of FDs, ONs, ON-FDs and their associates with GNPs. Designations: ln(*K_L_*^obs^)-logarithm of the Langmuir constant of the associate (ON/GNPs and ON-FD/GNPs); *n*^(obs)^-associate surface density; ln(*H*^obs^)-logarithm of the hydrophobicity coefficient of an ON and ON-FD; *Zd*^(calc)^-the total charge of the fluorophore and the linker between the FD and the ON; *pd*^(obs)^-passive diffusion of associates in an agarose gel without electric field; *Mr*^(calc)^-molecular weight of the FD and the linker between FD and the ON; *S*^(calc)^-fluorophore area; *d_H_*^(obs)^-hydrodynamic diameter of associate; ***ζ***^(obs)^-associate surface potential; ***ε***-fluorophore extinction coefficient; *μ*^(obs)^-normalized electrophoretic mobility of associate; *λex* (*λem*)-fluorophore excitation (emision) wavelength; *bl*^(obs)^-blurring of the main band of an associate in an agarose gel in the presence of an electric field.

Correlation analysis of experimental data and an array of published physicochemical characteristics of fluorophores used in this work showed a high degree of mutual correlation of all characteristics (Figure 5).

The mutual correlation was expected, given the structural similarity of planar fluorophores with polyaromatic compounds with a large π-system and, as a consequence, with a high content of unsaturated conjugated double bonds. An increase in the mass of such molecules means an increase in their surface area. An increase in the area of the π-system correlates with an increase in the efficiency of light absorption by fluorophores, a shift in the wavelength of the absorption maximum and, as a consequence, the maximums of excitation and emission of fluorescence of such residues in the long-wavelength part of the visible spectrum. For a series of xanthene-type dyes (Flu and RhB), the same effect is produced by a change in the exocyclic substituent. Considering these data, it is extremely difficult to single out those of the above characteristics that determine the affinity of the FD to the GNP surface. In such cases, the expansion of the experimental samples set by including ON-FDs with significantly different structures is necessary, and allowing the identification of structural factors that significantly affect the affinity of ON-FDs to GNPs. We included in the regression analysis characteristics of ONs with different lengths and sequences (Table 2).

An important result of the correlation analysis was the identification of a significant effect of the FD charge on the affinity of ON-FDs to the GNP surface. Comparison of the charge (*Zd*) value of FD, and the values of the *K_L_* for all ON-FDs and Flu-(D)k-Tm, showed a high correlation of these values. The cluster with high *K_L_* values (average *K_L_* = 0.051 nM^−1^) comprises a group of Flu-containing ONs which have *Zd* = −2 on the FD under the weakly acidic conditions used in the study (freshly synthesized GNPs stabilized with 4 mM citrate, pH ~5.5). Electrically neutral FDs (Pyr, Per and RhB, *Zd* = −1) comprise a cluster with an intermediate affinity for GNPs (average *K_L_* = 0.025 nM^−1^). The cluster with the highest affinity (mean *K_L_* = 0.01 nM^−1^) includes ON-FDs carrying positively charged fluorophores (*Zd* = 0). This pattern cannot be called completely expected, since fluorophore charge makes only an insignificant contribution relative to the charge of phosphodiester residues in oligonucleotide chains of the used series. That is, freshly synthesized GNPs with a high negative charge (Figure 5), provided by the surface layer of citrate anions, tend to more firmly fix ON-FDs, the fluorophore charge of which tends towards positive values. Thus, it is the structure of FD that determines the nature of this interaction; this indicates the realization of contact interactions between gold atoms and the terminal aromatic residue.

### 3.8. Regression Analysis

We tried to determine the possibility to predict the values of the K_L_ for the formation of ON-FD/GNPs, using the minimal set of selected characteristics from the collected dataset of reference and experimental characteristics of the FD residues and corresponding FD-ONs. Taking into account that the logarithm of *K_L_* (ln(*K_L_*)) is an energetic term and characterizes the free binding energy (Δ*G*⁰ at 298 K) of ONs and ON-FDs with GNPs, we tried to find reliable parameters, the additive contributions of which make it possible to calculate *K_L_* with a good accuracy using the quantitative structure activity relationship method (QSAR) [46]. This method allows us to develop a mathematical model describing the energy characteristics of ONs and ON-FDs binding with GNPs, and find (if they exist) statistically significant correlations between the structure of ONs and ON-FDs. The QSAR method is widely used for the search of biologically active compounds, and for the development of algorithms for predictively calculating the properties of nucleic acids [47,48].

Previously, we successfully used Multiple Regression Analysis (MRA) (one of the options for the search of statistically significant increments) to create an algorithm for calculating the hybridization properties of ONs when forming duplex structures of various types [49]. If a successful algorithm is found here, this means that the experimental *K_L_* values for different ONs and ON-FDs ($K_{Li}^{obs}$) correlate well with the corresponding calculated values ($K_{Li}^{calc}$), obtained by Equation (8):

(8) $$\ln(K_{L}^{calc})=\sum_{j}^{m}a_{j}\times F_{ji}$$

where *m*–limit number of j-type contributions, $F_{ji}$–value of a certain type of contribution, established experimentally or through independent calculations, for a specific ON-FD*_i_*, *j–*index characterizing the type or nature of the contribution, *i–*index designating certain type of FD and $a_{j}$–coefficient of proportionality that converts the value of the contribution $F_{ji}$ to the energy term of the sum $\ln(K_{L}^{calc})$ = $\sum_{j}^{m}a_{j}\times F_{ji}$.

We tried to establish the minimum set of contributions of the presented parameters (Table 3), taking into account their mutual correlation (Figure 5), giving preference to those types of characteristics ($F_{ji}$), the values of which can be estimated based on basic approaches or obtained experimentally. In total, the analyzed set included the characteristics of twenty-four ONs and their FD-labeled derivatives.

**Figure 6:**
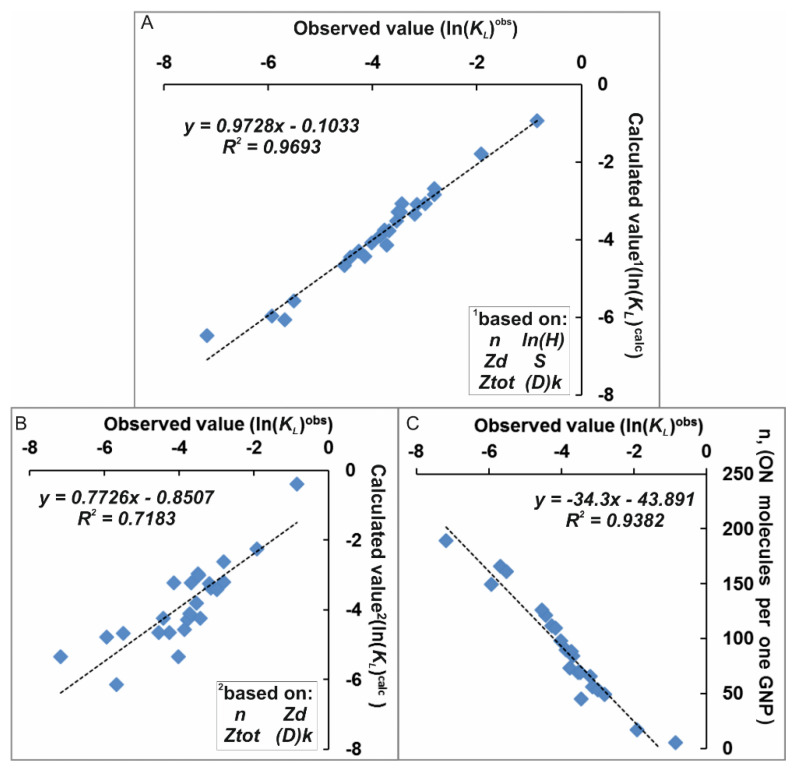
Correlation between (**A**,**B**) values of $\ln(K_{Li}^{obs})$ and $\ln(K_{Li}^{calc})$; (**C**) the values of $\ln(K_{Li}^{calc})$ and surface density of associates (n) based on factors (shown in the picture).

The best correlation (*R^2^* = 0.969) of the values $\ln(K_{Li}^{obs})\;\mathrm{vs}.\;\ln(K_{Li}^{calc})$ was observed when using six factors, which were significant for calculating the parameters of affinity of ON-FDs to GNP. The set of factors included surface density of associates (*n*); hydrophobicity of ON or ON-FD (ln(*H*)); charge of FD (*Zd*); surface area of FD (*S*); total charge of ON or ON-FD (*Ztot*); and number of aliphatic dodecyl residues ((D)k)) (Table 4, Figure 6A).

One of most significant characteristics was the surface density of associates (*n*) (determined experimentally under the same standardized conditions), since there was a fairly high correlation (*R^2^* = 0.938) between the values $\ln(K_{Li}^{obs})$ and $\ln(K_{Li}^{calc})$ (Figure 6B). If we exclude *n_i_* and *Hi* from consideration and restrict ourselves to a set of four parameters (ln(*H*), *Zd*, *Ztot* and (D)k), we find that these parameters introduce statistically significant contributions to the description of observed quantities $\ln(K_{Li}^{obs})$. Although in this case the correlation $\ln(K_{i}^{obs})\;\mathrm{vs}.\;\ln(K_{i}^{calc})$ was much lower (*R*^2^ = 0.718. Figure 6C) than in the case of usage of surface density (*n*), the fact of its presence indicates that there are structural factors that significantly affect the affinity of the ON to GNPs.

We have determined the minimum set of factors and the corresponding proportionality coefficients, which allow us to predict the efficiency of non-covalent adsorption of ON-FDs on GNPs. The results of our work allow us to conclude that in order to evaluate the K_L_, it is necessary (i) to determine the maximum surface density of ONs on one GNP under given conditions; and to characterize the relative hydrophobicity of the ONs bound with different residues; and (ii) to calculate the surface area of the FD, calculate the value of the charge of ON-FD and the value of the charge localized on fluorophore + linker (data required for such a calculation can be easily obtained from the formula of the used ONs). It should be noted that the above-mentioned experiments are simple to carry out.

**Figure 7:**
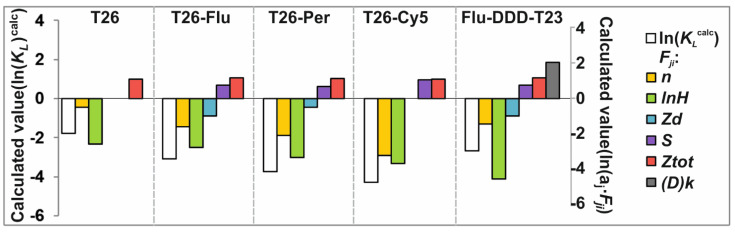
Graphical representation of energy contributions of $\ln(K_{L}^{calc})$, calculated based on the proportionality coefficients $a_{j}\;$ (Table 4) and factors $F_{ji}\;$(Table 3).

Thus, we established six factors (Table 4). An important element of our analysis (Equation (8)) is the proportionality coefficients that reflect the nature and relative contribution to the non-covalent interactions of ON or ON-FD with the GNP surface (Figure 7).

The results of MRA indicate that an increase in the number of sorptive molecules, its hydrophobicity and the value of the negative charge of terminal residue of the fluorophore-linker help to stabilize the associate. On the contrary, an increase in the total negative charge of the adsorbed ON and an increase in the FD area hinder its adsorption on GNPs. The presence of hydrophobic aliphatic residues (D)k in the composition of labeled ON has the same negative effect on its adsorption to GNPs. This observation additionally indicates that it is the presence of FD, with its π-system and exocyclic substituents, that is a crucial factor affecting the nature of the interaction of ON-FD and GNPs.

**Figure 8:**
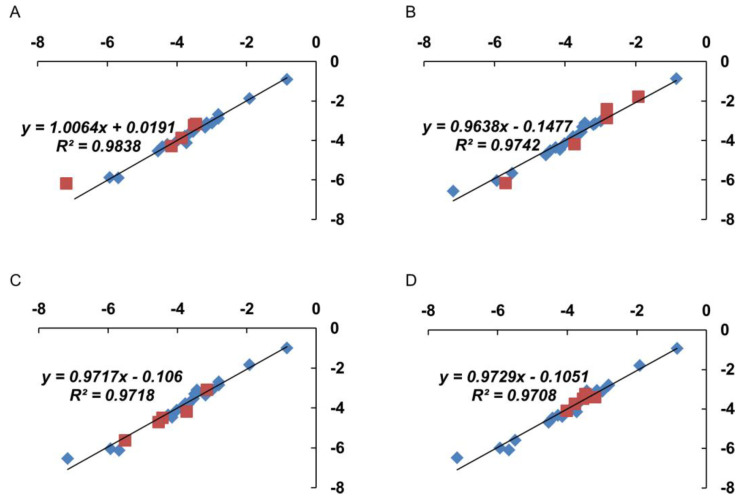
Cross-correlation between the observed ($\ln(K_{Li}^{obs})$) and calculated ($\ln(K_{Li}^{calc})$) values obtained by analysis of truncated samples of ONs. Blue color designates ONs of analyzed truncated set. Red color designates predicted values of the ONs and ON-FDs excluded from the set. Excluded: (**A**) T26-RdB, N*1-Flu, C26-Flu, T26-Pyr, T26-Cy7; (**B**) T26, T26-Cy7.5, Flu-T26, Flu-DDD-T23, A26-Pyr; (**C**) T26-Cy3, Flu-T26, A26-Pyr, A26-Cy5, C26-Cy5; (**D**) T26-Per, T26-Cy5.5, Flu-DD-T24, N*-Flu, N*2-Flu. X axis–experimental values of $\ln(K_{Li}^{obs})$, *Y* axis–calculated values of $\ln(K_{Li}^{calc})$.

To check the validity of the proposed approach using six factors, we additionally analyzed the predictive ability of the suggested algorithm for calculating ln(*K_L_*) values. We randomly formed four truncated sets, which included 19, not 24, ONs. The MRA results of the truncated sets are shown in the Figure 8.

Summarizing the data obtained, we can state that a change in the set of ONs and ON-FDs used for analysis does not lead to a significant change in the values of the obtained proportionality coefficients a_j_ for any of the considered factors $F_{ji}$.

Thus, it is possible to say that the algorithm based on Equation (8), using the identified significant structural factors and functional characteristics, can become the initial platform for the development of oligonucleotides with an increased affinity for GNPs during the formation of the corresponding non-covalent associates.

## 4. Discussion

To understand the fine mechanisms of various ONs and GNPs coupling to form non-covalent associates, it is worth understanding some structural features of associate components, starting from a primary one–GNPs, stabilized with citrate ions (Table S2). Molecular modeling showed that citrate ions do not form an unbroken layer, but are heterogeneously distributed over the surface of readily polarizable GNPs (crystal lattice (111 101)), and can migrate in the plane of the surface [13]. This means the presence of loci of a positive electric field potential on the GNP surface, and these loci are facing the environment. Thus, in the initial state, citrate-stabilized GNPs are highly charged NPs, having the loci on the surface, the positive charge of which specifies their affinity for negatively charged molecules.

Assuming that on the surface of citrate-stabilized GNPs there are loci with an increased positive electrostatic potential, shielded by an easily exchangeable layer of counterions, it is reasonable to suppose that such surface areas will become the primary center of attraction for ON-FDs. In addition, the amphiphilicity of GNPs allows them to actively bind molecules due to the implementation of nonspecific hydrophobic interactions. The surface of spherical GNPs of 12.7 + 2.0 nm in diameter is over 51.000 Å^2^. Under the condition of a single-layer planar arrangement of the FD π-system, GNPs can carry up to 300 compact Pyr residues, or no more than 200 residues of the largest polymethine Cy7.5 (according to our estimates).

The ONs are polyanions in their nature, and the introduction of one FD through a phosphate-containing linker is unable to drastically change the total charge of the resulting ON-FD molecule. The interaction of ON and ON-FD with GNPs stabilized by citrate seems to be extremely energetically unfavorable from the point of view of the electrostatic interactions. However, the aforementioned positively charged loci on the GNP surface can interact with anions of certain structural types, which therefore can bind with the surface of GNPs.

Residue FDs in ON-FDs, when they begin to contact with the GNP surface, could be involved both in electrostatic and other interactions of a non-electrostatic nature. One example of these interactions is represented by the hydrophobic interactions between the aromatic system of FD and GNP. We observed that an increase in the hydrophobicity of ON-FDs, coupled with an increase in size of the fluorophore π-system, promoted an increase in the affinity of ON-FDs for GNPs (this was not true in the case of an increase in the number of hydrophobic aliphatic dodecyl amine (D)k residues).

The difference in energy contributions from the total charge of the molecule (*Ztot*) and the charge (*Zd*) of the FD residue, also leads to the hypothesis that the presence of aromatic FD is the factor that largely determines the affinity of ON-FD to GNP. The FD in ON-FD increases the probability of initiation complexes (ON-FD~GNP) formation, which, as a result, promotes the fixation of ON-FDs on the GNP surface.

Obviously, the efficiency of the interaction is largely determined by the probability of formation of an initiation complex at the first stage of formation of any intermolecular or molecular/supramolecular structure (GNP is one of the supramolecular structures). For example, for NA/NA binding, the increased efficiency of formation of GC-rich duplexes is determined not only by the stability of the resulting double helix, but also by the increased probability of initiation complex formation in comparison with the cases of the formation of AT-rich secondary structures [50,51].

The probability of contact of NP with an ON-FD molecule is most likely controlled by the significant electrostatic repulsion of equal-charged interacting components of the sorptive/solvent system.

**Figure 9:**
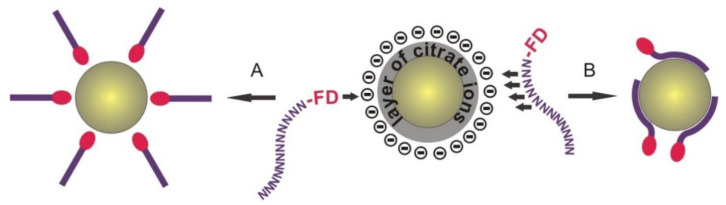
Simplified scheme of the mechanism of formation of non-covalent associates of ON-FD/GNP type. A, B–different pathways, corresponding to models of one–(A) or multi-point (B) interaction of fluorophore-containing DNA with GNP. Citrate ions are absent in the images of the resulting associates for better perception.

The linear nature of ON allows us to assume that the phosphodiester chain of ON, approaching to GNP, will be orthogonally oriented relative to the GNP surface. In this case, the properties of the terminal fluorophore residue can play a decisive role, because it can serve both as a factor of the orientation of the ON-FD molecule relative to the GNP surface, and as an “anchor” binding ON-FD and GNPs (Figure 9). This means that, in our opinion, pathway A of initiation of non-covalent adsorption of ON-FDs on GNPs (Figure 9) is much more prevailing than the alternative pathway B.

Having considered all the factors that determine the initial state of interacting components in the system of ON-FD and GNP, we can make an assumption about the mechanism of the formation of non-covalent associates of ON-FD/GNP type, and about the role of the FD in this process. Taken together, our study revealed that the presence of FD residues containing aromatic systems is a factor that largely determines the affinity of ON-FD for GNP.

## 5. Conclusions

We carried out, for the first time, a systemic study concerning the effect of FD structure (in the composition of ON-FD) on the efficiency of the formation of their non-covalent associates with GNPs. Oligonucleotides of various lengths and nucleotide composition, as well as the corresponding ON-FDs (FDs from the series of xanthene dyes, polymethine dyes or dyes based on polycyclic aromatic hydrocarbons), were used.

The surface density of associates; hydrophobicity of ON-FDs; charge of FDs; total charge of ON or ON-FDs; and surface area of FDs were revealed as the key factors determining the affinity of native or FD-labeled ONs to GNPs. These factors comprise a specific set, which makes it possible to predict with high reliability the efficiency of ONs and ON-FDs binding with GNPs. The principal possibility of creating an algorithm for the predictive calculation of the efficiency of interaction of ONs of a certain structural group with GNPs was demonstrated. For the first time, a hypothetical model has been proposed that describes the mechanism of contact interaction between negatively charged nano-objects, such as citrate-stabilized GNP, and ONs or their ON-FDs derivatives.

The studies performed are important because they reveal the prospects for the directed design of ligands based on oligonucleotide derivatives capable of forming non-covalent associates with metal nanoparticles, and also demonstrate the methodology and fundamental principles of conducting research of this kind.

## Figures and Tables

**Table 1:** Langmuir constants (*K_L_*), equilibrium dissociation constant (*K_D_*) for T26-FD/GNPs and surface density (*n*) of T26-FDs.

ON-FD	*K_L_* (nM^−1^)	*n* (Molecules per Particle)	*K_D_* * (nM)
T26 (without FD)	0.147 ± 0.0510	17 ± 5	-
T26-Pyr	0.032 ± 0.0110	45 ± 13	0.181 ± 0.074
T26-Per	0.023 ± 0.0120	73 ± 5	-
T26-Flu	0.043 ± 0.0100	56 ± 4	0.224 ± 0.021
T26-RhB	0.021 ± 0.0050	90 ± 4	-
T26-Cy3	0.012 ± 0.0020	121 ± 4	-
T26-Cy5	0.014 ± 0.0020	112 ± 4	0.064 ± 0.005
T26-Cy5.5	0.018 ± 0.0030	98 ± 2	-
T26-Cy7.5	0.003 ± 0.0003	166 ± 2	-
T26-Cy7	0.00077 ± 0.00004	189± 1	-

*–*K_D_* values measured for 5′-end-[^32^P]-ONs.

**Table 2:** Langmuir constants of ON-FD/GNPs and the surface density of ONs on one particle.

**ON-FD**	Sequence (*5′ → 3′)*	*K_L_* (nM^−1^)	*n* (Molecules per Particle)
*-Flu	GATATGATGACGTTAGTTAG-Flu	0.0286 ± 0.0056	69 ± 7
N*1-Flu	***TTTTTT***GATATGATGACGTTAGTTAG-Flu	0.0312 ± 0.0074	69 ± 5
N*2-Flu	***TTGTTG***GATATGATGACGTTAGTTAG-Flu	0.0300 ± 0.0065	70 ± 4
FAM-D-T25	Flu-D-TTTTTTTTTTTTTTTTTTTTTTTTT	0.0503 ± 0.0175	54 ± 2
FAM-DD-T24	Flu-DD-TTTTTTTTTTTTTTTTTTTTTTTT	0.0410 ± 0.0143	66 ± 2
FAM-DDD-T23	Flu-DDD- TTTTTTTTTTTTTTTTTTTTTTT	0.0600 ± 0.0243	49 ± 1
A26-FITC	AAAAAAAAAAAAAAAAAAAAAAAAAA-Flu	0.0251 ± 0.0060	84 ± 4
A26-Pyr	AAAAAAAAAAAAAAAAAAAAAAAAAA-Pyr	0.0240 ± 0.0072	89 ± 3
A26-Cy5	AAAAAAAAAAAAAAAAAAAAAAAAAA-Cy5	0.0106 ± 0.0025	126 ± 1
C26-FITC	CCCCCCCCCCCCCCCCCCCCCCCCCC-Flu	0.0157 ± 0.0036	110 ± 1
C26-Cy5	CCCCCCCCCCCCCCCCCCCCCCCCCC-Cy5	0.0041 ± 0.0005	161 ± 4
T40	TTTTTTTTTTTTTTTTTTTTTTTTTTTTTTTTTTTTTTTT	0.4299 ± 0.1270	5 ± 3
T6	TTTTTT	0.0026 ± 0.0004	149 ± 16

***Green type*** indicates additional nucleotide fragment to N*-Flu; D–hydrophobic non-nucleotide insert containing dodecyl amine residue (Figure S3).

**Table 3:** Parameters used for Multiple Regression Analysis.

	ln(*K_L_*^obs^)	ln(*K_L_*^calc^)	Std. Err.	*n*	ln(*H*^obs^)	*Zd*	*Mr*	*S*	Ztot	(D)k	5′End Dye	Dye
T26	−1.917	−1.788	0.149	17	1.403	0	0	0	−25	0	0	0
T40	−0.844	−0.936	0.224	5	1.408	0	0	0	−39	0	0	0
T6	−5.934	−5.961	0.234	149	1.367	0	0	0	−5	0	0	0
T26-Pyr	−3.442	−3.063	0.132	45	1.812	−1	466	399	−26	0	0	1
T26-Per	−3.772	−3.742	0.089	73	1.822	−1	744	446	−26	0	0	1
T26-RdB	−3.863	−3.908	0.090	90	1.892	−1	695	736	−26	0	0	1
T26-Flu	−3.147	−4.449	0.086	56	1.503	−2	584	513	−27	0	0	1
T26-Cy3	−4.423	−4.291	0.093	121	1.902	0	647	642	−25	0	0	1
T26-Cy5	−4.269	−4.069	0.143	112	2.003	0	673	710	−25	0	0	1
T26-Cy5.5	−4.017	−3.089	0.093	98	2.177	0	773	818	−25	0	0	1
T26-Cy7	−7.169	−6.463	0.139	189	2.177	0	739	797	−25	0	0	1
T26-Cy7.5	−5.684	−6.056	0.117	166	2.378	0	826	903	−25	0	0	1
Flu-T26	−2.813	−2.836	0.096	49	1.461	−2	456	513	−27	0	1	1
Flu-D-T25	−2.990	−3.070	0.091	54	1.899	−2	559	513	−27	1	1	1
Flu-DD-T24	−3.194	−3.345	0.140	66	2.245	−2	662	513	−27	2	1	1
Flu-DDD-T23	−2.814	−2.686	0.211	49	2.474	−2	765	513	−27	3	1	1
N*-Flu	−3.540	−3.511	0.103	69	1.412	−2	456	513	−21	0	0	1
N*1-Flu	−3.507	−3.281	0.098	70	1.400	−2	456	513	−27	0	0	1
N*2-Flu	−3.474	−3.279	0.096	69	1.415	−2	456	513	−27	0	0	1
A26-Flu	−3.686	−3.771	0.102	84	1.470	−2	584	513	−27	0	0	1
A26-Pyr	−3.729	−4.139	0.078	89	1.780	−1	466	399	−26	0	0	1
A26-Cy5	−4.543	−4.656	0.090	126	2.004	0	673	710	−25	0	0	1
C26-Flu	−4.152	−4.424	0.135	110	1.470	−2	584	513	−27	0	0	1
C26-Cy5	−5.504	−5.576	0.119	161	2.012	0	673	710	−25	0	0	1

Where ln(*K_L_*^obs^)–logarithm of observed *K_L_* of associate; ln(*K_L_*^calc^)–logarithm of calculated K_L_ of associate; Std.Err.–standard error; *n*–associate surface density; ln(*H*^obs^)–logarithm of relative hydrophobicity of each ON and ON-FD; *Zd*–charge of FD + linker fragment; *Mr*–mass of FD + linker fragment; *S*–surface area of FD accessible for solvent (Å^2^); *Ztot*–total charge of ON or ON-FD; (D)k–number of aliphatic dodecyl residues; 5′-end dye–presence of FD on the 5′-end of ON; dye–presence of FD on the any end of ON.

**Table 4:** List of statistically significant factors and corresponding values of proportionality coefficients determined by Multiple Regression Analysis.

**Factors, *F j***	$a_{j}$	Std. Err.	*p*-Level
*n*	−0.02598	0.00214	4.1 × 10^−10^
ln(*H*)	−1.66333	0.28448	1.5 × 10*^−^*^5^
*Zd*	0.44722	0.07271	8.3 × 10*^−^*^6^
*S*	0.00136	0.00033	7.2 × 10*^−^*^4^
*Ztot*	−0.03948	0.01328	8.1 × 10*^−^*^3^
(D)k	0.61519	0.13007	1.7 × 10*^−^*^4^

$a_{j}$–proportionality coefficients in Equation (8).

## Data Availability

Data is available on request from the corresponding author.

## Supplementary Materials

The following are available online at https://www.mdpi.com/article/10.3390/nano11051178/s1, Table S1: Name, color* and structure of fluorophores, and linkers, connecting a fluorophore with an oligonucleotide; experimental and reference characteristics of these compounds, Table S2: Hydrodynamic diameter (*d_H_*), surface charge (ζ) and normalized electrophoretic mobility (*µ*) of T26-FD/GNPs, Figure S1: Gold nanoparticles (citrate stabilized) adsorbed on formvar film for 30 sec. Transmission electron microscopy., Figure S2: Nonlinear regression (binding curve). Radiolabeled T26-Cy5 (0.03 nM) was titrated with GNPs (0.03–3 nM), Figure S3: Structure of Flu-(D) kTm. D–a saturated dodecyl residue based on a phosphodiester of an N-substituted diethanolamine fragment; (*k* = 1, 2 or 3), *m* = 26-k–amount of D-residues Figure S4: (A) Optical absorption spectra of T26-FD/GNPs (solid curves) and T26-FD (dashed curves), (B) enlarged part of the spectrum, Figure S5: (A) HPLC profiles of T26 and T26-FD in an acetonitrile concentration gradient from 0 to 50% on an Agilent 1200 Series using a Zorbax 5 μm Eclipse-XDB-C18 80 Å column (150 × 4.6 mm^2^), (B) dependence of GNP coverage with T26 and T26-FDs on their hydrophobicity (column exit time), Figure S6: Dependence of the GNP coverage with ONs on their hydrophobicity.

## Abbreviations

ON	oligodeoxynucleotide
GNP	gold nanoparticle
ON/GNP	non-covalent associate–nanoconstruct obtained by non-covalent binding (synonyms: interaction, adsorption) of ON and GNP
ON-GNP	covalent conjugate of ON and GNP–nanoconstruct obtained by covalent binding (synonym: immobilization) of ON and GNP
FD	fluorescent dye (fluorescent label)
ON-FD	ON labeled with FD
*ε*	FD molar coefficient of absorption
*n*	surface density number of ONs attached to surface of one GNP
RP HPLC	reversed-phase high-performance liquid chromatography
obs	observed (or experimental) value
calc	calculated value
ref	reference data
*K_L_*	Langmuir constant of the associate
*K_D_*	Equilibrium Dissociation Constant of the associate
*tr*	hydrophobicity or retention time of ONs or ON-FDs
*H*	hydrophobicity coefficient of ONs or ON-FDs
*Zd*	the total charge of the FD and the linker between the FD and the ON
*pd*	passive diffusion of non-covalent associates in an agarose gel without electric field
*bl*	blurring of the main band of an associate in an agarose gel in the presence of an electric field
*Mr*	molecular weight of the ONs or ON-FDs
*S*	fluorophore area
*d_H_*	hydrodynamic diameter of non-covalent associate
*ζ*	associate surface potential
*μ*	normalized electrophoretic mobility of associate in the agarose gel
*λex (λem)*	fluorophore excitation (emission) wavelength
IOD	integrated optical density
(D)k	number (k) of aliphatic dodecyl residues (D)

## References

[1] Busatto S., Pham A., Suh A., Shapiro S., Wolfram J. (2019). Organotropic drug delivery: Synthetic nanoparticles and extracellular vesicles. Biomed. Microdevices.

[2] Zhou W., Wang F., Ding J., Liu J. (2014). Tandem phosphorothioate modifications for DNA ad-sorption strength and polarity control on gold nanoparticles. ACS Appl. Mater. Interfaces.

[3] Chen N., Wan Y., Liu H., Su Y., Fan C., Wei M., Li F., Huang Q., Pei H. (2012). Designed diblock oligonucleotide for the synthesis of spatially isolated and highly hybridizable functionalization of DNA–gold nanoparticle nanoconjugates. J. Am. Chem. Soc..

[4] Chen N., Wei M., Sun Y., Li F., Pei H., Li X., Su S., He Y., Wang L., Shi J. (2014). Self-assembly of poly-adenine-tailed CpG oligonucleotide-gold nanoparticle nanoconjugates with immunostimulatory activity. Small.

[5] Yao G., Pei H., Li J., Zhao Y., Zhu D., Zhang Y., Lin Y., Huang Q., Fan C. (2015). Clicking DNA to gold nanoparticles: Poly-adenine-mediated formation of monovalent DNA-gold nanoparticle conjugates with nearly quantitative yield. NPG Asia Mater..

[6] Zhang X., Liu B., Dave N., Servos M.R., Liu J. (2012). Instantaneous attachment of an ultrahigh density of nonthiolated DNA to gold nanoparticles and its applications. Langmuir.

[7] Lu W., Wang L., Li J., Zhao Y., Zhou Z., Shi J., Zuo X., Pan D. (2015). Quantitative investigation of the poly-adenine DNA dissociation from the surface of gold nanoparticles. Sci. Rep..

[8] Zhang X., Liu B., Servos M.R., Liu J. (2013). Polarity control for nonthiolated DNA adsorption onto gold nanoparticles. Langmuir.

[9] Kumar A., Mandal S., Mathew S.P., Selvakannan P.R., Mandale A.B., Chaudhari R.V., Sastry M. (2002). Benzene- and anthracene-mediated assembly of gold nanoparticles at the liquid-liquid interface. Langmuir.

[10] Curry D., Cameron A., MacDonald B., Nganou C., Scheller H., Marsh J., Beale S., Lu M., Shan Z., Kaliaperumal R. (2015). Adsorption of doxorubicin on citrate-capped gold nanoparticles: Insights into engineering potent chemotherapeutic delivery systems. Nanoscale.

[11] Li-na M.A., Dian-Jun L.I.U., Zhen-Xin W. (2010). Synthesis and Applications of Gold Nanoparticle Probes. Chin. J. Anal. Chem..

[12] Oh J., Park D.H., Joo J.H., Lee J. (2015). Recent advances in chemical functionalization of nano-particles with biomolecules for analytical applications. Anal. Bioanal. Chem..

[13] Perfilieva O.A., Pyshnyi D.V., Lomzov A.A. (2019). Molecular Dynamics Simulation of Polarizable Gold Na-noparticles Interacting with Sodium Citrate. J. Chem. Theory Comput..

[14] Sapsford K.E., Algar W.R., Lorenzo B., Gimmill K.B., Casey B.J., Oh E., Stewart M.H., Medintz I.L. (2013). Functionalizing nanoparticles with biological molecules developing chemis-tries that facilitate nanotechnology. Chem. Rev..

[15] Barnaby S.N., Perelman G.A., Kohlstedt K.L., Chinen A.B., Schatz G.C., Mirkin C.A. (2016). Design Considerations for RNA Spherical Nucleic Acids (SNAs). Bioconjug. Chem..

[16] Zhang X., Servos M.R., Liu J. (2012). Surface science of DNA adsorption onto citrate-capped gold nanoparticles. Langmuir.

[17] Epanchintseva A., Vorobjev P., Pyshnyi D., Pyshnaya I. (2018). Fast and Strong Adsorption of Native Oligonucleotides on Citrate-Coated Gold Nanopar-ticles. Langmuir.

[18] Epanchintseva A., Dolodoev A., Grigor’eva A., Chelobanov B., Pyshnyi D., Ryabchikova E., Pyshnaya I. (2018). Non-covalent binding of nucleic acids with gold nanoparticles provides their stability and effective desorption in environment mimicking biological media. Nanotechnology.

[19] Poletaeva J., Dovydenko I., Epanchintseva A., Korchagina K., Pyshnyi D., Apartsin E., Ryabchikova E., Pyshnaya I. (2018). Non-covalent associates of siRNAs and AuNPs enveloped with lipid layer and doped with amphiphilic pep-tide for efficient siRNA delivery. Int. J. Mol. Sci..

[20] Epanchintseva A., Poletaeva J., Pyshnyi D., Ryabchikova E., Pyshnaya I. (2019). Long-term stability and scale-up of noncovalently bound gold nanoparticle-siRNA suspensions. Beilstein J. Nanotechnol..

[21] Vorobjev P., Epanchintseva A., Lomzov A., Tupikin A., Kabilov M., Pyshnaya I., Pyshnyi D. (2019). DNA Binding to Gold Nanoparticles through the Prism of Molecular Selection: Sequence—Affinity Relation. Langmuir.

[22] Sun W., Lu Y., Mao J., Chang N., Yang J., Liu Y. (2015). Multidimensional sensor for pattern recognition of proteins based on DNA-gold nanoparticles conjugates. Anal. Chem..

[23] Liu J. (2012). Adsorption of DNA onto gold nanoparticles and graphene oxide: Surface science and applications. Phys. Chem. Chem. Phys..

[24] Pylaev T.E., Volkova E.K., Kochubey V.I., Bogatyrev V.A., Khlebtsov N.G. (2013). DNA detection assay based on fluorescence quenching of rhodamine B by gold nano-particles: The optical mechanisms. J. Quant. Spectrosc. Radiat. Transf..

[25] Shashkova V.V., Epanchintseva A.V., Vorobjev P.E., Razum K.V., Ryabchikova E.I., Pyshnyi D.V., Pyshnaya I.A. (2017). Multilayer associates based on oligonucleotides and gold nanoparticles. Russ. J. Bioorg. Chem..

[26] Giusti W.G., Adriano T. (1993). Synthesis and characterization of 5’-fluorescent-dye-labeled oli-gonucleotides. PCR Methods Appl..

[27] Astakhova I.K., Wengel J. (2013). Interfacing Click Chemistry with Automated Oligonucle-otide Synthesis for the Preparation of Fluorescent DNA Probes Containing Internal Xan-thene and Cyanine Dyes. Chem. A Eur. J..

[28] Borer P.N., Fasman T.E. (1975). Optical properties of nucleic acids, adsorption, and circular dicroism spectra. Handbook of Biochemistry and Molecular Biology: Nucleic Acids.

[29] Glen Research: Oligonucleotide Synthesis Supplies & Supports. https://www.glenresearch.com.

[30] Laser Photomedicine and Biomedical Optics at the Oregon Medical Laser Center. http://www.omlc.org.

[31] Thermo Fisher Scientific. https://www.thermofisher.com.

[32] Vailaya A., Horva´th C. (1996). Retention Thermodynamics in Hydrophobic In-teraction Chromatography. Ind. Eng. Chem. Res..

[33] Vailaya A. (2005). Fundamentals of Reversed Phase Chromatography: Thermodynamic and Exothermodynamic Treatment. J Liq. Chrom. Relat. Tech..

[34] Wei Tsai C., Yih Chen W., Chyu Ruaan R. (2012). Retention Prediction of Peptide Diastereomers in Reversed-Phase Liquid Chromatography Assisted by Molecular Dynamics Simulation. Langmuir.

[35] Frens G. (1973). Controlled Nucleation for the Regulation of the Particle Size in Monodisperse Gold Suspensions. Nat. Phys. Sci..

[36] Liu X., Atwater M., Wang J., Huo Q. (2007). Extinction coefficient of gold nanoparticles with different sizes and different capping ligands. Colloids Surf. B Biointerfaces.

[37] Ryder S.P., Recht M.I., Williamson J.R., Lin R.J. (2008). Quantitative Analysis of Protein-RNA Interactions by Gel Mobility Shift. RNA-Protein Interaction Protocols. Methods in Molecular Biology.

[38] Golyshev V.M., Abramova T.V., Pyshnyi D.V., Lomzov A.A. (2018). A new approach to precise thermodynamic characterization of hybridization properties of modified oligonucleotides: Comparative studies of deoxyribo- and glycine morpholine pentaadenines. Biophys. Chem..

[39] Amaya-González S., López-López L., Miranda-Castro R., de-los-Santos-Álvarez N., Miranda-Ordieres A.J., Lobo-Castañón M.J. (2015). Affinity of aptamers binding 33-mer gliadin peptide and gluten proteins: Influence of immobilization and labeling tags. Anal. Chim. Acta.

[40] Tuleuova N., Jones C.N., Yan J., Ramanculov E., Yokobayashi Y., Revzin A. (2010). Development of an Aptamer Beacon for Detection of Interferon-Gamma. Anal. Chem..

[41] Kang K.A., Wang J., Jasinski J.B., Achilefu S. (2011). Fluorescence Manipulation by Gold Nanoparticles: From Complete Quenching to Extensive Enhancement. J. Nanobiotechnol..

[42] Borovok N., Gillon E., Kotlyar A. (2012). Synthesis and Assembly of Conju-gates Bearing Specific Numbers of DNA Strands per Gold Nanoparticle. Bioconjugate Chem..

[43] Kupryushkin M.S., Nekrasov M.D., Stetsenko D.A., Pyshnyi D.V. (2014). Ef-ficient Functionalization of Oligonucleotides by New Achiral Nonnucleosidic Monomers. Org. Lett..

[44] Pavlova A.S., Dovydenko I.S., Kupryushkin M.S., Grigor’eva A.E., Pyshnaya I.A., Pyshnyi D.V. (2020). Amphiphilic “Like-A-Brush” Oligonucleotide Conjugates with Three Dodecyl Chains: Self-Assembly Features of Novel Scaffold Compounds for Nucleic Acids Delivery. Nanomaterials.

[45] Markov O.V., Filatov A.V., Kupryushkin M.S., Chernikov I.V., Patutina O.A., Strunov A.A., Chernolovskaya E.L., Vlassov V.V., Pyshnyi D.V., Zenkova M.A. (2020). Transport Oligonucleotides—A Novel System for Intracellular Delivery of Antisense Therapeutics. Molecules.

[46] Verma J., Khedkar V.M., Coutinho E.C. (2010). 3D-QSAR in Drug Design—A Review. Curr. Top Med. Chem..

[47] Santa Lucia J. (2018). How much free energy is absorbed upon breaking DNA base pairs? Comment on “DNA melting and energetics of the double helix” by Maxim Frank-Kamenetskii. Phys. Life Rev..

[48] Santa Lucia J., Hicks D. (2004). The thermodynamics of DNA structuralmotifs. Annu. Rev. Biophys. Biomol. Struct..

[49] Lomzov A.A., Pyshnyi D.V. (2012). Considering the oligonucleotide secondary structures in thermodynamic and kinetic analysis of DNA duplex formation. Biophysics.

[50] Gu X.-B., Nakano S., Sugimoto N. (2007). Consecutive GC base pairs determine the energy barrier of DNA duplex formation under molecularly crowded conditions. Chem. Commun..

[51] Lin M.-C., Macgregor R.B. (1997). Activation Volume of DNA Duplex Formation. Biochemistry.

