# Supplementary material for: Effect of Fluorescent Labels on DNA Affinity for Gold Nanoparticles
Source: Nanomaterials (Basel). 2021 Apr 29;11(5):1178. doi: 10.3390/nano11051178 (PMC8145642; doi:10.3390/nano11051178)
Supplement: Supplementary file 1 [file nanomaterials-11-01178-s001.zip › nanomaterials-1186310-supplementary.pdf]

Supplementary Material

Effect of Fluorescent Labels on DNA Affinity for Gold Nanoparticles

Anna V. Epanchintseva, Ekaterina A. Gorbunova, Elena I. Ryabchikova *, Inna A. Pyshnaya and Dmitrii V. Pyshnyi *

Institute of Chemical Biology and Fundamental Medicine, SB RAS, Lavrentiev ave. 8, 630090 Novosibirsk, Russia; annaepanch@niboch.nsc.ru (A.V.E.); gorbunova-ekaterina@inbox.ru (E.A.G.); pyshnaya@niboch.nsc.ru (I.A.P.)

* Correspondence: lenryab@niboch.nsc.ru (E.I.R.); pyshnyi@niboch.nsc.ru (D.V.P.); Tel.: +8-383-3635153 (E.I.R.); +8-383-3635151 (D.V.P.)

Citation: Epanchintseva, A.V.; Gorbunova, E.A.; Ryabchikova, E.I.; Pyshnaya, I.A.; Pyshnyi, D.V. Effect of Fluorescent Labels on DNA Affinity for Gold Nanoparticles. *Nanomaterials* **2021**, *11*, 1178. https://doi.org/10.3390/nano11051178

Academic Editor: Paolo Pengo

Received: 31 March 2021
Accepted: 27 April 2021
Published: 29 April 2021

Publisher's Note: MDPI stays neutral with regard to jurisdictional claims in published maps and institutional affiliations.

Copyright: © 2021 by the authors. Submitted for possible open access publication under the terms and conditions of the Creative Commons Attribution (CC BY) license (http://creativecommons.org/licenses/by/4.0/).

Table S1. Name, color* and structure of fluorophores, and linkers, connecting a fluorophore with an oligonucleotide; experimental and reference characteristics of these compounds.

FD	Fluorofor Dye	Linker between FD and ON	*Zd* FD	*Mr* FD-linker (a.u.)	*S* FD (A^2)	λex^1, λex^2 (nm)	λem^1, λem^2 (nm)	ε^2 FD (l × mol/cm^1)
Pyr	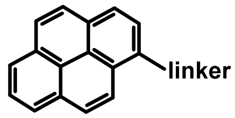	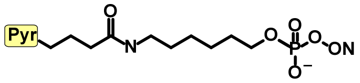	−1	466	399	323<br>326 [4,5]	378<br>377 [4,6]	54,000 [3]
Per	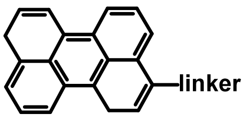	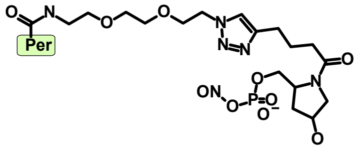	−1	744	446	422<br>435 [4,7]	490<br>467 [4,8]	36,000 [4]
Flu	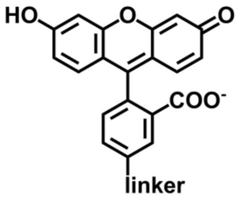	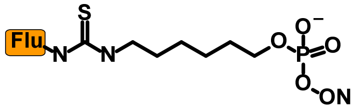<br>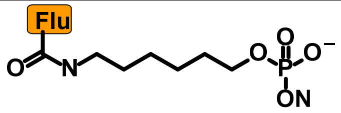	−2	584	513	489<br>492 [10]	519<br>518 [10]	70,000 [9]
RhB	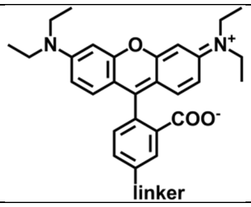	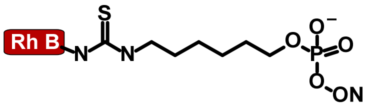	−1	695	736	557<br>543 [10]	581<br>580 [10]	106,000 [3]
Cy3	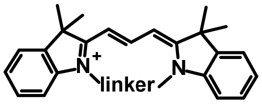	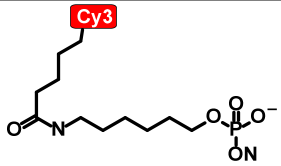	0	647	642	545<br>555 [4]	566<br>570 [4]	150,000 [4]
Cy5	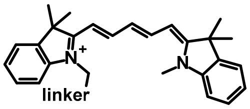	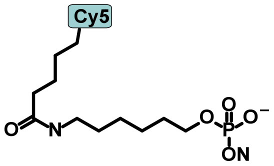	0	673	710	649<br>646 [4]	663<br>662 [4]	250,000 [4]
Cy5.5	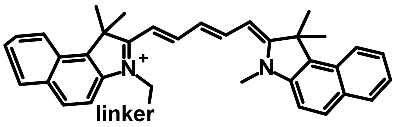	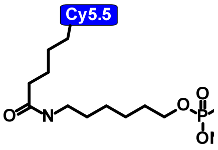	0	773	818	684<br>684 [4]	704<br>710 [4]	250,000 [4]
Cy7	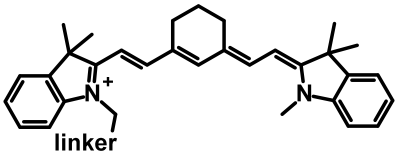	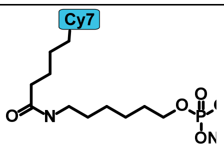	0	739	797	750<br>750 [4]	773<br>773 [4]	199,000 [4]
Cy7.5	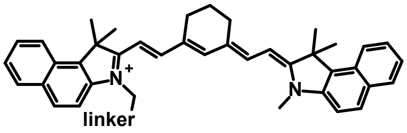	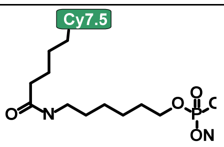	0	826	903	750 [11]<br>788 [4]	809<br>808 [4]	223,000 [4]

* - color of FD solution in water at pH 5.5; [1] – experimental data; [2] – reference data; [3] – www.omlc.org (information source); [4] – www.lumiprobe.com (information source); [5] – closest reference value to experimentally obtained of λex (343, 326, 313. 276, 265, 242, 234 nm); [6] – closest reference value of λem (397, 377 nm) to experimentally obtained; [7] – closest reference value of λex (435, 408, 252 nm) to experimentally obtained; [8] – closest reference value of λem (476, 439 nm) to experimentally obtained; [9] – www.thermofisher.com (information source); [10] – www.sigmaaldrich.com (information source); [11] – 750 nm – maximum possible λex in ClarioStar Plus.

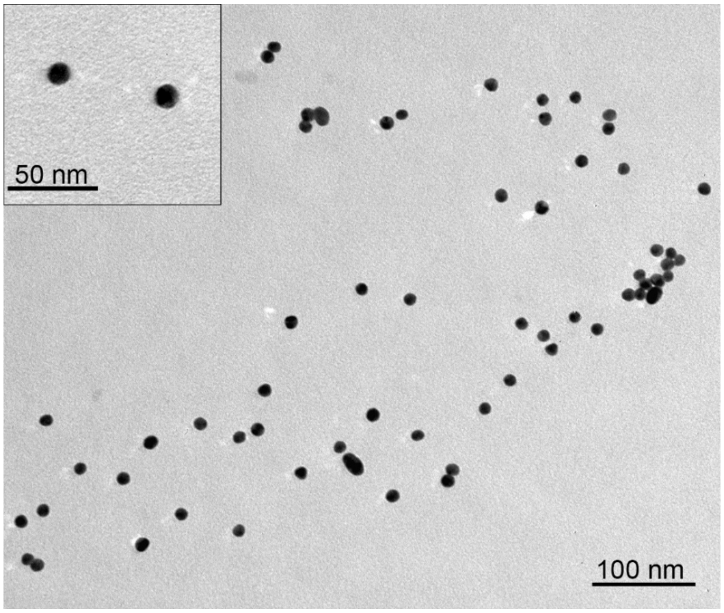

Figure S1. Gold nanoparticles (citrate stabilized) adsorbed on formvar film for 30 sec. Transmission electron microscopy.

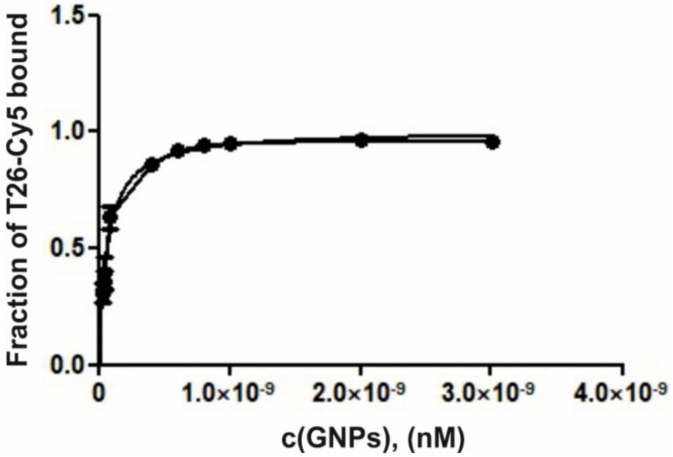

Figure S2. Nonlinear regression (binding curve). Radiolabeled T26-Cy5 (0.03 nM) was titrated with GNPs (0.03–3 nM). Four measurements were made for each point, standard deviation is presented in the form of error bars. Data were fitted to the equation $Y = B_{max}X/(K_D + X)$, where X is the total concentration of GNPs, and Y is the bound fraction of T26-Cy5, B_{max} is the number of binding sites. Parameters obtained are $K_D = 0.064 \pm 0.005$ nM, $B_{max} = 1.003 \pm 0.017$, $R^2 = 0.94$.

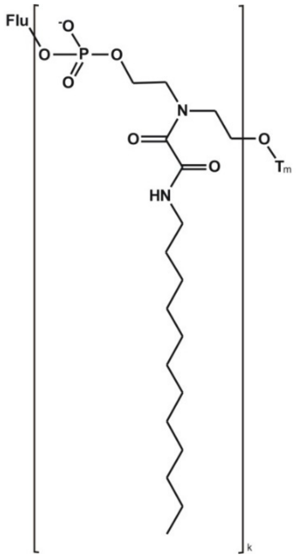

Figure S3. Structure of Flu-(D) kTm. D - a saturated dodecyl residue based on a phosphodiester of an N-substituted diethanolamine fragment; (k = 1, 2 or 3), m = 26 − k - amount of D-residues.

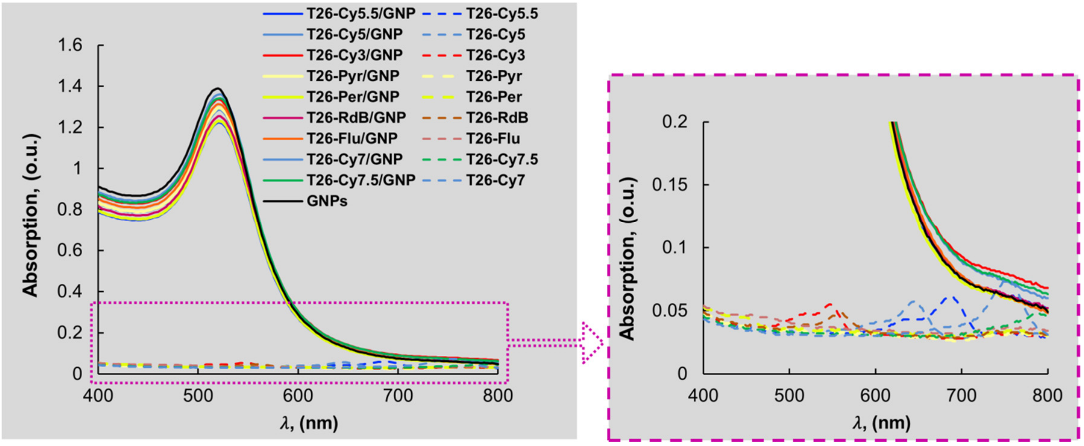

(**A**) (**B**)

Figure S4. (**A**) Optical absorption spectra of T26-FD/GNPs (solid curves) and T26-FD (dashed curves). (**B**) Enlarged part of the spectrum. Concentrations of associates and T26-FD are equal.

Table S2. Hydrodynamic diameter (d_H), surface charge (ζ) and normalized electrophoretic mobility (μ) of T26-FD/GNPs.

ON-FD	d_H (nm)	ζ (mV)	μ *
T26(without dye)/GNP	26.5 ± 0.3	−30.5 ± 1.6	1
T26-Pyr/GNP	40.6 ± 1.3	−37.4 ± 3.1	0.88
T26-Per/GNP	30.3 ± 0.3	−35.9 ± 0.9	0.83
T26-Flu/GNP	29.7 ± 0.9	−35.6 ± 1.0	0.94
T26-RhB/GNP	32.4 ± 1.3	−35.8 ± 2.2	0.86
T26-Cy3/GNP	30.0 ± 0.5	−33.2 ± 1.4	0.89
T26-Cy5/GNP	29.8 ± 1.3	−35.3 ±0.7	0.87
T26-Cy5.5/GNP	32.3 ± 0.5	−51.1 ± 0.5	0.85
T26-Cy7.5/GNP	28.6 ± 0.2	−44.3 ± 1.4	0.86
T26-Cy7/GNP	27.3 ± 0.2	−44.6 ± 2.6	0.87

* – Electrophoresis was carried out for 10–90 min at 5 V/cm in 25 mM Tris, 250 mM glycine buffer, pH 8.3.

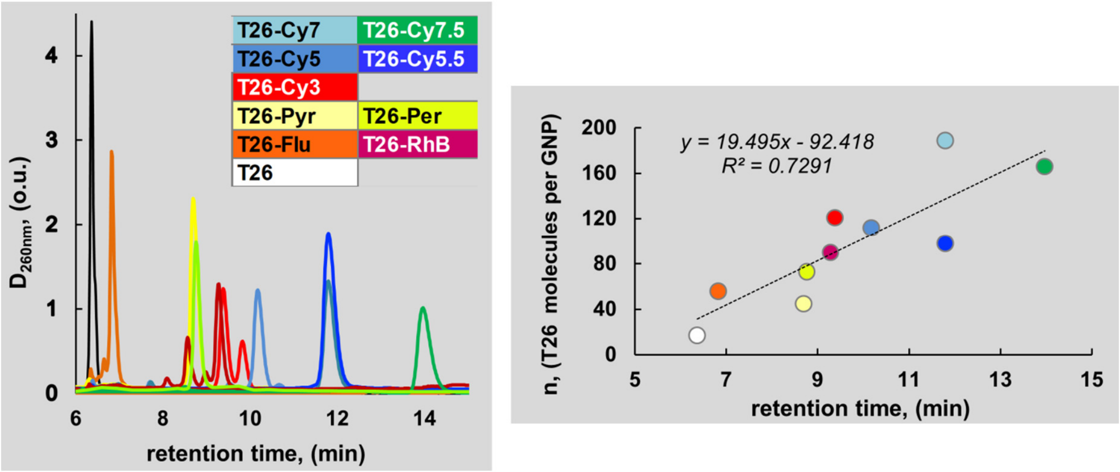

(**A**) (**B**)

Figure S5. (**A**) HPLC profiles of T26 and T26-FD in an acetonitrile concentration gradient from 0–50% on an Agilent 1200 (Santa Clara, CA, USA) Series using a Zorbax 5 µm Eclipse-XDB-C18 80 Å column (150 × 4.6 mm^2). (**B**) Dependence of GNP coverage with T26 and T26-FDs on their hydrophobicity (column exit time).

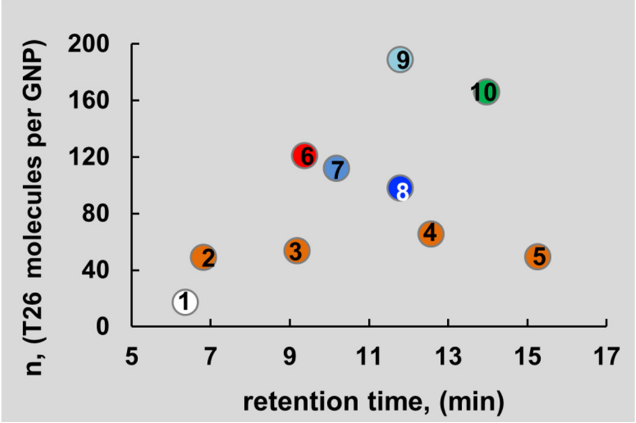

Figure S6. Dependence of the GNP coverage with ONs on their hydrophobicity, where (1) – T26, (2) Flu-T26, (3) - Flu-D-T25, (4) - Flu-DD-T24, (5) - Flu-DDD-T23, (6) – T26-Cy3, (7) – T26-Cy5, (8) – T26-Cy-5.5, (9) – T26-Cy7, (10) – T26-Cy7.5.
